# Multi‐locus genome‐wide association study for grain yield and drought tolerance indices in sorghum accessions

**DOI:** 10.1002/tpg2.20505

**Published:** 2024-09-10

**Authors:** Yirgalem Tsehaye, Temesgen M. Menamo, Fetien Abay, Taye Tadesse, Kassahun Bantte

**Affiliations:** ^1^ Department of Dryland Crop and Horticultural Sciences, College of Dryland Agriculture and Natural Resources Mekelle University Mekelle Ethiopia; ^2^ Tigray Agricultural Research Institute Mekelle Ethiopia; ^3^ Department of Horticulture and Plant Science, College of Agriculture and Veterinary Medicine Jimma University Jimma Ethiopia; ^4^ Ethiopian Institute of Agricultural Research Addis Ababa Ethiopia

## Abstract

Drought is a significant factor that causes yield loss in essential cereal crops such as sorghum [*Sorghum bicolor* (L.) Moench], necessitating the development of drought‐tolerant varieties adaptable to various water conditions. This study aimed to pinpoint drought‐tolerant sorghum lines and genomic regions for tolerance by utilizing 216 sorghum accessions in stressed and non‐stressed environments at two locations. Genetic diversity was evident among accessions in terms of grain yield under different watering regimes. Drought stress indices such as the stress tolerance index, mean productivity, geometric mean productivity, harmonic mean productivity, yield stability index, and yield index were identified as effective measures for selecting drought‐tolerant sorghum. Cluster analysis classified genotypes into four groups based on their association with grain yield, highlighting Acc. #28546 and Acc. #216739 as highly drought tolerant across environments. This study identified 32 and 22 quantitative trait nucleotides (QTNs) for drought indices and grain yield under stress and non‐stress conditions, respectively, at two locations, with five common QTNs linked to multiple drought indices. Colocation analysis revealed that these QTNs were associated with known stay‐green‐related quantitative trait loci (QTLs), and 47 putative genes near these QTNs potentially influenced drought tolerance traits. It is suggested that accession selection considers multiple indices for robust evaluation. Understanding the identified genes and their functions provides insights into the genetic mechanisms governing plant responses to drought stress, offering prospects for developing improved drought‐resistant sorghum varieties through further genetic research.

## Core Ideas

- Drought‐tolerant sorghum lines and genomic regions were identified using stress indices.
- Acc. #28546 and Acc. #216739 lines were the most tolerant.
- The majority of quantitative trait nucleotides (QTNs) colocalized with priori quantitative trait loci (QTLs) related to stay‐green.
- Colocated genes offer insights for breeding resilient varieties.

**Abbreviations**
KM: kinship matrix
ML‐GWAS: multi‐locus genome‐wide association studies
mrMLM.GUI: multi‐locus random‐SNP‐effect mixed linear model with graphical user interface
PC: principal components
QTN: quantitative trait nucleotide

## 1 INTRODUCTION

Among cereal grains, sorghum [*Sorghum bicolor* (L.) Moench] is an important cereal crop that ranks fifth in global production (FAOSTAT, 2022). It is known for its resilience to harsh environmental conditions, particularly drought stress, making it a valuable crop in regions with limited water availability (Hadebe et al., 2017). While drought conditions pose significant challenges, the crop's very adaptable (Assefa et al., 2020). As such, understanding the genetic basis of drought tolerance in sorghum is of paramount importance for developing improved varieties that can thrive under water‐limited conditions. To evaluate the response of genotypes to drought stress, several selection indices based on the mathematical relationship between stress and optimum conditions have been proposed for selecting genotypes based on their performance in stressful and non‐stressed environments. These selection indices include but are not limited to the stress tolerance index (STI) (Fernandez, 1992), stress tolerance (Rosielle & Hamblin, 1981), mean productivity (MP) (Rosielle & Hamblin, 1981), geometric mean productivity (GMP), stress susceptibility index (SSI) (Fischer & Maurer, 1978), yield index (YI) (Gavuzzi et al., 1997), yield reduction ratio (YR) (Golestani Araghi & Assad, 1998), harmonic mean (HM) (Jafari et al., 2009), drought resistance index (Schneider et al., 1997), and yield stability index (YSI) (Bouslama & Schapaugh, 1984). Each of these indices represents a different aspect of the mathematical formula used to indicate drought stress tolerance, and understanding the genetic control of these indices can provide insights into the underlying mechanisms of drought tolerance in sorghum.

Genome‐wide association studies (GWAS) have emerged as powerful tools for dissecting the genetic architecture of complex plant traits, including drought tolerance (Menamo et al., 2023; Xin et al., 2022). GWAS enable the identification of genetic variants associated with specific traits by examining the correlation between single nucleotide polymorphisms (SNPs) and phenotypic variation across a diverse panel of genotypes (Maina et al., 2022). Multi‐locus genome‐wide association studies (ML‐GWAS) have gained attention for their ability to capture the combined effects of multiple loci on complex traits, providing a more comprehensive understanding of the genetic basis of polygenic traits such as drought tolerance in sorghum (Zhong et al., 2021).

In recent years, several studies have focused on unraveling the genetic determinants of drought tolerance traits in sorghum using ML‐GWAS (Duresso et al., 2023; Enyew et al., 2022; Wondimu et al., 2023). These studies have employed diverse sorghum germplasm collections, high‐throughput genotyping platforms, and precise phenotyping protocols to determine the genetic basis of drought tolerance at a fine scale. By integrating genotypic and phenotypic data from large sorghum populations, researchers have identified key genomic regions associated with different drought tolerance traits (Maina et al., 2022; X. Wang et al., 2020; Xin et al., 2022; Y. Zhang et al., 2023), shedding light on the genetic pathways and molecular mechanisms underlying drought adaptation in sorghum. In particular, Xin et al. (2022) identified several genome‐wide associations between plant height and MP, relative drought indices, and stress tolerance indices.

One of the main challenges in dissecting the genetic basis of drought tolerance indices in sorghum is the polygenic nature of these traits (Bernardo, 2008). Drought tolerance is controlled by a complex network of genes, each contributing to different aspects of the plant's response to water stress (Mwadzingeni et al., 2016). ML‐GWAS offer a promising approach to address this complexity by simultaneously considering multiple genomic loci and their interactions, thus capturing the combined effects of multiple genes on drought tolerance indices (S.‐B. Wang et al., 2016). Moreover, the availability of high‐density SNP markers and advanced statistical methods for ML‐GWAS has enabled researchers to accurately detect small‐effect loci and uncover epistatic interactions contributing to drought tolerance in sorghum. However, limited studies have been performed on ML‐GWAS using drought indices. This fine‐scale dissection of the genetic architecture of drought tolerance indices can provide breeders with valuable information for marker‐assisted selection and genomic prediction, facilitating the development of sorghum varieties with improved drought resilience. In this context, this study aimed to provide information on the identification of drought‐tolerant accessions and genomic regions associated with drought tolerance‐based drought tolerance indices of sorghum grain yield. The study pinpointed genomic regions linked to stay‐green traits that enhance drought adaptation after flowering.

## 2 MATERIALS AND METHODS

### 2.1 Description of the study area

The field experiment was conducted at two sites at the Ethiopia Institute of Agricultural Research: the Melkassa Agricultural Research Center (MARC) and the Werer Agricultural Research Center (WARC). The MARC is situated at a latitude of 8° 24′ 985″ N and a longitude of 39° 19′ 185″ E, with an altitude of 1550 m above sea level. The WARC is located at a latitude of 9° 22″ N and a longitude of 40° 11″ E at an altitude of 750 m above sea level. Both areas are recognized as semiarid and drought prone regions. These sites were selected for the study due to their historical weather data indicating low rainfall, in addition to the presence of well‐organized irrigation facilities.

### 2.2 Climatic conditions during the crop growth period

**FIGURE 1:**
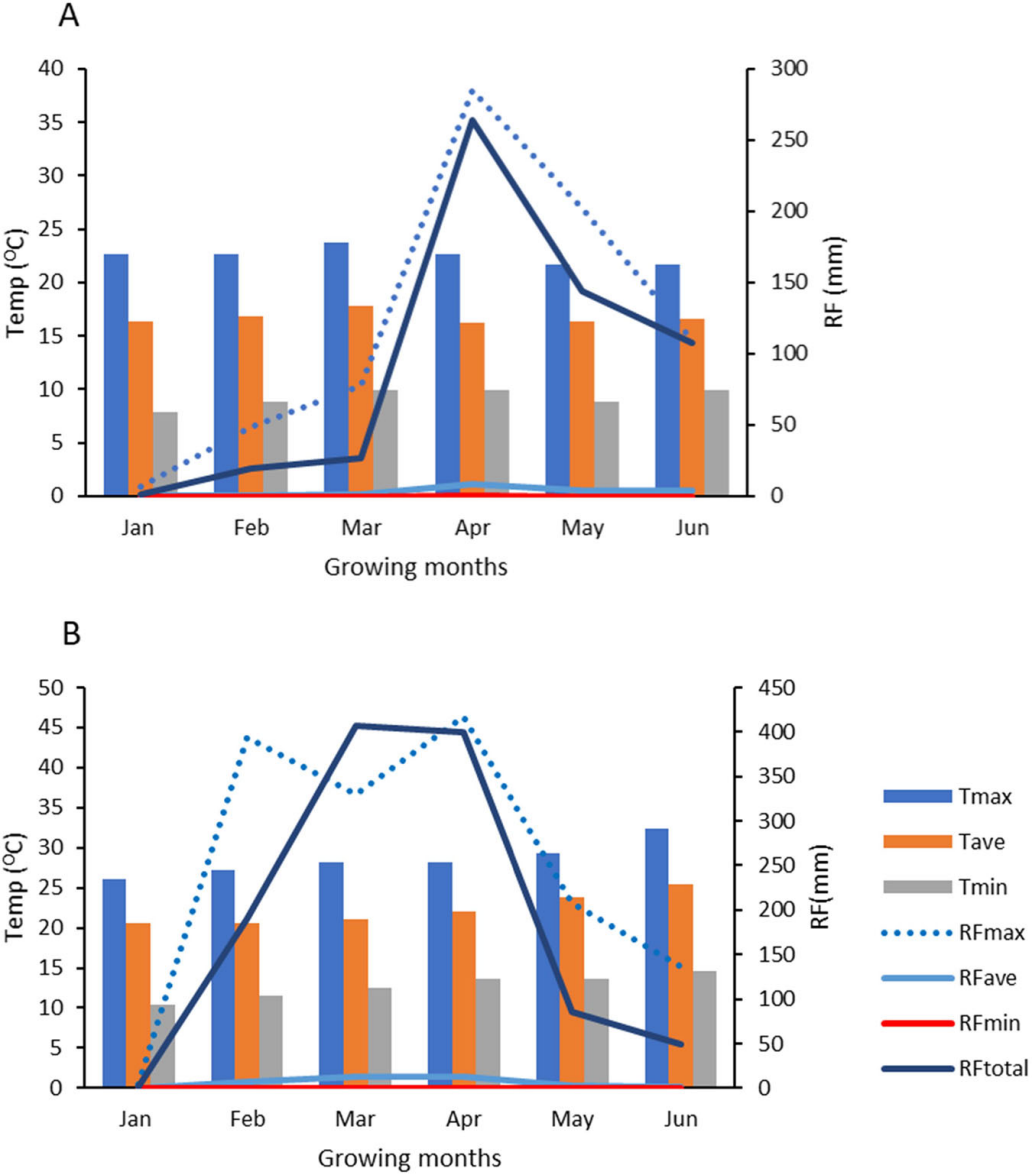
The monthly temperature (°C) and rainfall (mm) during the study period at Werer (A) and Melkassa (B). RF, rainfall; RFave, monthly average rainfall; RFmax, monthly maximum rainfall; RFmin, monthly minimum rainfall; RFtotal, monthly total rainfall; Tave, monthly average temperature; Tem, temperature; Tmax, monthly maximum temperature; Tmin, monthly minimum temperature.

The experiment was conducted between January and June in 2019. At the Melkassa location, the total monthly rainfall ranged from 0.89 to 263.47 mm, with daily values fluctuating between a minimum of 0.0 mm and a maximum of 37.99 mm in April (Figure 1A). The highest temperature recorded at this site varied from 21.71°C in January to a peak of 23.68°C in March. Conversely, at the Werer site, the total monthly rainfall ranged from 0.62 to 399.55 mm, with the highest temperature variations ranging from 26.15°C in January to a maximum of 32.43°C in June (Figure 1B).

**Core Ideas**

- Drought‐tolerant sorghum lines and genomic regions were identified using stress indices.
- Acc. #28546 and Acc. #216739 lines were the most tolerant.
- The majority of quantitative trait nucleotides (QTNs) colocalized with priori quantitative trait loci (QTLs) related to stay‐green.
- Colocated genes offer insights for breeding resilient varieties.

### 2.3 Genetic materials

A total of 216 (212 accessions and four varieties) genetic materials were used (Table S1). These materials were selected based on yield performance and key drought adaptation traits such as less leaf senescence, flowering time, and stay‐greenness. The materials were originally obtained from the International Crops Research Institute for the Semi‐Arid Tropics (ICRISAT) and Ethiopian Biodiversity Institute via the National Sorghum Improvement Research Project of the Ethiopian Institute of Agricultural Research (EIAR) at the Melkassa Agricultural Research Center.

### 2.4 Experimental design, treatments, and trial management

The field experiment was conducted using a row–column design (8 × 27) with two replications to compare well‐watered and water‐stressed growth conditions. It was carried out during the 2019 postrainy season (January to June), creating two contrasting soil moisture regimes by adjusting the frequency of irrigation. The genetic materials were planted in three batches. Batches were planted 15 days apart with the late maturity accessions (≥80 days to flowering) planted first, then mid‐maturity (70–80 days to flowering), and finally early maturity accessions (55–69 days to flowering) (Table S1). The seeds were directly drilled into single rows 5 m in length with a spacing of 75 cm between rows. Two weeks after sowing, the plants were thinned to an interplant spacing of 15 cm (30 plants per row). Fertilizers were applied at the recommended rates of 50 kg/ha urea (46% N) and 100 kg/ha diammonium phosphate (DAP; 18% N and 46% P_2_O_5_). Half of the urea and all the DAP were applied at the time of sowing. The remaining urea was top‐dressed at the six‐ to eight‐leaf stage. All recommended management practices (weeding, cultivation, etc.) were uniformly applied under both growing conditions. Furrow irrigation was used for irrigation. Initially, the entire field received equal irrigation up to the flowering stage (from January to April). The irrigation was applied once or twice a week based on climatic condition of the experimental sites. However, following flowering (May to June), the drought‐stressed plants were deprived of irrigation water (water stressed) to induce post‐flowering drought. In contrast, the plants in the well‐watered treatment groups continued to receive irrigation after flowering once or twice per week to prevent drought stress at any stage. This rainfall was not enough for proper seed blooming to the grain filling stage. With respect to early grain filling, sorghum uses approximately 0.35 in. (8.89 mm) of water per day or 266.7 mm per month (Stichler & Fipps, 2003).

### 2.5 Data collection

#### 2.5.1 Grain yield

Grain yield (kg/ha) was taken from two plots and the plot data were converted to per hectare according to the International Board for Plant Genetic Resources (IBPGR) and ICRISAT descriptors (IBPGR & ICRISAT, 1993).

#### 2.5.2 Genotypic data

The experiment involved sowing seeds of different genotypes in a greenhouse at the Melkassa Agricultural Research Center using cell trays. After 10 days, leaf samples were collected from three seedlings of each genotype, and the pooled leaf samples were frozen using a freeze dryer and stored at −80°C before being sent to the Biosciences Eastern and Central Africa‐International Livestock Research Institute (BecA‐ILRI) Hub in Nairobi.

Genomic DNA (gDNA) was then extracted from the frozen tissues following the cetyltrimethylammonium bromide protocol with some modifications (Rogers & Bendich, 1985). The gDNA samples were subsequently subjected to DNA sequencing through genotyping by sequencing technology using Diversity Arrays Technology Sequence technology. This process was carried out via the Integrated Genotype Service and Support platform in Nairobi, Kenya, utilizing a combination of diversity arrays technology (DArT) complexity reduction methods and next‐generation sequencing protocols described in the works of Elshire et al. (2011) and Kilian et al. (2012). The complexity reduction method involved digestion with the methylation‐sensitive restriction enzyme PstI, along with the use of frequently cutting enzymes such as AluI, BstNI, TaqI, or MseI. Polymerase chain reaction (PCR) adapters were subsequently ligated to the PstI fragment ends, followed by PCR amplification using primers complementary to the PstI adapters. Only fragments with PstI adapters at both ends were amplified. DNA fragments were digested, ligated to adapters, and amplified via PCR (Kilian et al., 2012). Sequencing was performed on an Illumina HiSeq 2000 platform using a single‐read strategy with 77 cycles. The resulting sequence data were analyzed using the DArT analytical pipelines (Barilli et al., 2018). The primary pipeline employed stringent quality control measures, filtering out low‐quality sequences based on barcode region characteristics. Unique sequences per sample were then used for marker calling. Subsequently, the data were processed through the secondary pipeline, which utilized DArT P/L's proprietary SNP calling algorithms (DArTsoftseq). Finally, the SNP markers were identified with reference to the genome of Sorghum bicolor V3.1.

### 2.6 Data analysis

#### 2.6.1 Analysis of phenotypic data

The grain yield analysis of variance was performed using the fit linear mixed‐effects models *lmer v1.1* function in the *lmerTest* v3.1 R package (Kuznetsova et al., 2017). Genotypes were fixed effects, and rows and columns were random effects. Broad‐sense heritability (*H* ^2^) was estimated using the formula suggested by Pariyar et al. (2021).

$$H^{2}=\frac{\sigma_{g}^{2}}{\sigma_{g}^{2}\;+\;\left(\sigma_{e}^{2}/n\right)},$$

where *σ* ^2^ *_g_* is the genotypic variance, *σ* ^2^ *_e_* is the phenotypic variance, and *n* is the number of replicates. *σ* ^2^g = (Msg − MSe)/*r*, where Msg are the mean square genotypes, MSe is the mean square error, and *r* are the replications.

The STI was calculated to identify germplasm accessions with high stress tolerance and overall good yield performance. The drought stress indices were calculated using nine indices (Table 1). Pearson correlation was estimated between grain yield and drought indices using *corr* function in RStudio. To perform the clustering, we used *cluster* v2.1.6 and *factoextra v1.0.7* R packages and distance between data point using Euclidean method, scaling the data, and constructed the dendrogram using ward.D2 method (Kassambara & Mundt, 2020). To assess the stability of the clusters, we performed 1000 bootstrap replicates. The number cluster was estimated using silhouette method in factoextra v1.0.7 R packages.

**TABLE 1:** Drought tolerance indices were calculated using the following equations.

	Index	Formula	Outcome	References
(i)	Stress susceptible index (SSI)	$\mathrm{SSI}=\frac{1\;-\frac{Y_{s}}{Y_{i}}}{1\;-\frac{{\bar{Y}}_{s}}{{\bar{Y}}_{i}}}$	The accessions with SSI < 1 are more resistant to drought stress conditions.	Fischer and Maurer (1978)
(ii)	Stress tolerance index (STI)	$\mathrm{STI}=\frac{Y_{i}\times \;Y_{s}}{{{\bar{Y}}_{i}}^{2}}$	The accessions with high STI values are tolerant to drought stress.	Fernandez (1992)
(iii)	Mean productivity (MP)	$\mathrm{MP}\;=\frac{Y_{i}+Y_{S}}{2}$	The accessions with high value MP are more desirable	Rosielle and Hamblin (1981)
(iv)	Tolerance (TOL)	TOL=Yi−Ys	The accessions with low values of the TOL index are more stable in two different conditions.	Rosielle and Hamblin (1981)
(v)	Geometric mean productivity (GMP)	$\mathrm{GMP}\;=\sqrt{Y_{i}\times Y_{s}}$	The accessions with high value of GMP index are more desirable.	Schneider et al. (1997)
(vi)	Harmonic mean productivity (HMP)	$\mathrm{HMP}=\frac{2(Y_{i}-Y_{s})}{\left(Y_{i}+Y_{s}\right)}$	The accessions with high value of HMP index are more desirable.	Jafari et al. (2009)
(vii)	Yield index (YI)	$\mathrm{YI}\;=\frac{Y_{s}}{{\bar{y}}_{s}}$	The accessions with high value of YI index are suitable for drought stress condition.	Gavuzzi et al. (1997)
(viii)	Yield reduction ratio (YR)	$\mathrm{YR}=1-Y_{s}/Y_{i}$	The accessions with low value of YR index are suitable for drought stress condition.	Golestani Araghi and Assad (1998)
(ix)	Yield stability index (YSI)	$\mathrm{YSI}\;=\frac{Y_{s}}{Y_{i}}$	The accessions with high YSI values are stable genotypes under stress and non‐stress conditions.	Fischer and Maurer (1978)

Abbreviations: Yi, yield mean of each accession under well‐watered conditions; ${\bar{Y}}_{i}$, grand mean yield of accessions under well‐watered conditions; Ys, yield mean of each accession under water stressed conditions; ${\bar{Y}}_{s}$, grand mean yield of accessions under water stressed conditions.

### 2.7 GWAS analysis

A total of 17,637 robust SNP markers obtained from DArTSeqTM data were employed for the GWAS analysis. The SNP dataset was carefully filtered to exclude SNPs with a minor allele frequency (MAF) less than 0.05 or missing values exceeding 25%. Missing values were further inferred using the Beagle 5.0 software package (Browning et al., 2018). Marker trait association analyses were conducted using six ML‐GWAS models, namely, multi‐locus random‐SNP‐effect mixed linear model (S.‐B. Wang et al., 2016), fast multi‐locus random‐SNP‐effect mixed linear model (Tamba & Zhang, 2018), fast multi‐locus random‐SNP‐effect EMMA (Wen et al., 2018), polygenic‐background‐control‐based least angle regression plus empirical Bayes (J. Zhang et al., 2017), polygenic‐background‐control‐based Kruskal–Wallis test plus empirical Bayes (Ren et al., 2018), and Iterative Sure Independence Screening EM‐Bayesian LASSO (Tamba et al., 2017), which were implemented in the “multi‐locus random‐SNP‐effect mixed linear model with graphical user interface (*mrMLM.GUI*)” v4.0.2 R package (Wen et al., 2018). The first five principal components (PC) were estimated using TASSEL v5 software (Bradbury et al., 2007) and added as population structure and kinship matrix (KM) was estimated using VanRaden method in “*mrMLM.GUI*” v4.0.2 R package (Wen et al., 2018). PC and KM were used to control population structure, and GWAS analysis with and without accounting for population structure and kinship significantly affected the identified associations and the overall robustness of our results (Armero et al., 2019). Quantitative trait nucleotides (QTNs) with a logarithm of the odds score of at least 3.00 (approximately −log10 = 4.0) were considered significantly associated with the traits (S.‐B. Wang et al., 2016). A two‐stage approach was employed. Initially, candidate markers were identified using multiple algorithms. Subsequently, these markers were integrated into a single model where effects were estimated using empirical Bayes. Finally, non‐zero effects are determined to be true QTNs through likelihood ratio testing. Although a less stringent significance threshold was utilized, these methods have high power and accuracy and a low false positive rate (Y.‐W. Zhang et al., 2020). Additionally, only SNP markers identified in at least three models were designated as reliable trait‐associated QTNs. Similarly, QTNs that were repeatedly detected in three or more models and demonstrated phenotypic variation (R2 > 10%) were designated major QTNs. The resulting −log10 (*p*) values obtained from the ML‐GWAS approaches were utilized to create Manhattan and *Q*‒*Q* plots using the *mrMLM.GUI* V4.0.12 package (Wen et al., 2018).

### 2.8 Identification of quantitative trait loci (QTLs) colocated with previously reported QTLs for drought tolerance

The colocation of the significant QTNs with previously identified QTLs was determined using the Sorghum QTL ATLAS database (Mace et al., 2019) within 65 kb of linkage disequilibrium (LD) decay reported by G. Girma et al. (2019) of significant QTNs detected in both locations or in at least three drought‐induced instances per location. The LD *r* ^2^ was estimated using TASSEL v5 software (Bradbury et al., 2007), and the decay was estimated using R software. Colocation was done on the consensus markers sourced from the Sorghumbase database v7 (Gladman et al., 2022).

### 2.9 Identification of candidate genes and functional annotation

Potential candidate genes were obtained from the genomic data portal for the plant family via the Phytozome BioMart tool (Goodstein et al., 2012). Initially, according to G. Girma et al. (2019), a window of 65 kb pairs was established upstream and downstream of each significant QTN to identify colocations with previously identified QTLs for drought tolerance. The functional annotations of the candidate genes were downloaded from Phytozome based on the database website, and the genetic information pertaining to the annotated functions of the resistance QTNs and their relevance to sorghum resistance to water stress was determined.

## 3 RESULTS

### 3.1 Variations in grain yield and drought indices

The analysis of variance revealed a highly significant difference (*p* < 0.01) in the grain yield among the genotypes across the two water treatments and location interactions (Table 2). The grain yield varied from 197.33 kg/ha (Acc. #220260) to 6341.33 kg/ha (Acc. #216736) under water‐stressed conditions and from 501.33 kg/ha (Acc. #220236) to 8394.66 kg/ha (Acc. #3583) under well‐watered conditions at Melkassa (Table 2; Table S2). In Werer, the grain yield varied from 585.0 kg/ha (Acc. #220013) to 5045.0 kg/ha (B‐35) under water stress conditions and from 990.0 kg/ha (Acc. #28688) to 6250 kg/ha (B‐35) under well‐watered growth conditions (Table 1; Table S2). Among the four check varieties, Melkam exhibited the greatest yield (4421.3 kg/ha) under water stress conditions at Melkassa (Table S2). The yields of four accessions (Acc. #220268, Acc. #234115, Acc. #216739, and Acc. #216736) were greater than those of Melkam under water stress conditions. In Werer, contrast, the B‐35 variety had the greatest yield (5045.0 kg/ha) compared to all the other accessions and varieties under water stress conditions (Table S2). The drought tolerance indices revealed variation among accessions regarding yield reduction (Table 2). The broad‐sense heritability estimate was high (>0.60) in both well‐watered and water‐stressed conditions at Werer, while it was high (>0.60) and moderate (0.30–0.60) at Melkassa in well‐watered and water‐stressed conditions, respectively.

**TABLE 2:** Summary of grain yield under stress and well‐watered conditions and drought induces values in both Melkassa and Werer.

	Melkassa				Werer			
Traits	Maximum	Minimum	Mean	*H* ^2^	Maximum	Minimum	Mean	*H* ^2^
Ys	6341.33	197.33	1742.39^a^	0.56	5045.00	585.00	1932.06^a^	0.84
Yi	8394.67	501.33	2995.92^a^	0.72	6250.00	990.00	3023.58^a^	0.72
SSI	0.50	−0.41	−0.02	–	2.29	0.00	0.95	–
STI	5.31	0.01	0.69	–	3.45	0.08	0.70	–
MP	6925.33	394.67	2369.15	–	5647.50	867.50	2477.82	–
GMP	6900.67	344.82	2228.09	–	5615.27	849.15	2385.31	–
HM	6876.09	301.26	2112.94	–	5583.22	831.18	2301.57	–
TOL	6928.00	10.67	1253.53	–	4295.00	5.00	1091.52	–
YSI	0.99	0.08	0.60	–	1.00	0.90	0.92	–
YR	0.92	0.01	0.40	–	0.83	0.00	0.34	–
YI	3.64	0.11	1.00	–	2.61	0.30	1.00	–

Abbreviations: GMP, geometric mean productivity; *H* ^2^, broad‐sense heritability; HMP, harmonic mean productivity; MP, mean productivity; SSI, stress susceptible index; STI, stress tolerance index; TOL, tolerance; YI, yield index; Yi, mean yield of each accession under well‐watered conditions; YR, yield reduction ratio; Ys, mean yield of each accession under water stressed conditions; YSI, yield stability index.

^a^ Highly significant (*p* < 0.01).

### 3.2 Correlation between grain yield and drought indices

**FIGURE 2:**
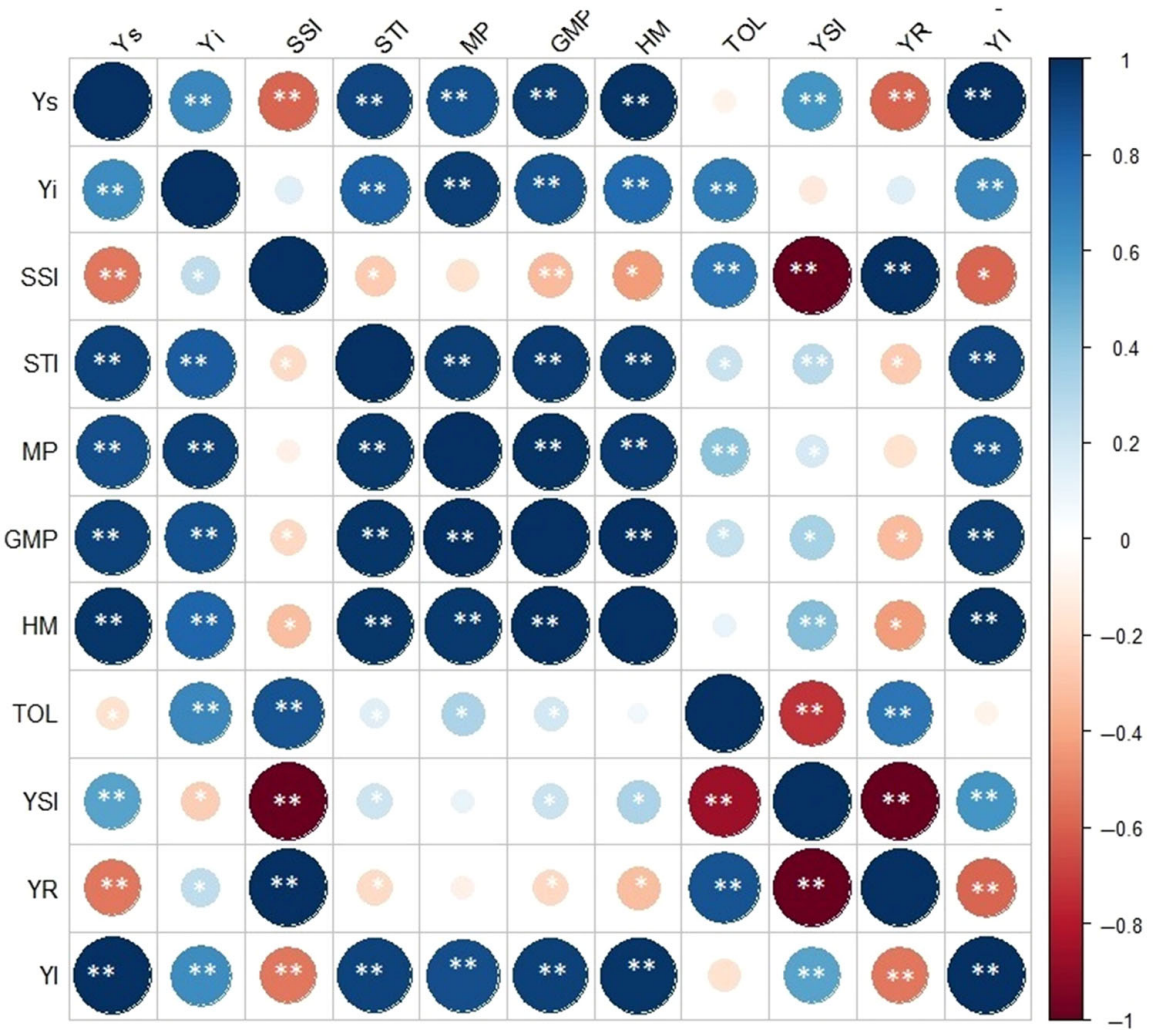
Spearman's rank correlation coefficients between grain yields and drought indices over two experimental sites (Melkasa above diagonal and Werer below diagonal) in sorghum accessions. The significance values were * = 5% and ** = 1% for the mean yield in well‐watered (Yi), mean yield in water stressed (Ys), stress susceptibility index (SSI), stress tolerance index (STI), mean productivity (MP), geometric mean productivity (GMP), harmonic mean productivity (HMP), tolerance index (TOL), yield stability index (YSI), yield reduction ratio (YR), and yield index (YI).

To identify the most effective drought tolerance index, it is essential to estimate the correlation coefficients between grain yield (Yi and Ys) and other quantitative indices of drought tolerance. A reliable index should exhibit a highly significant correlation with grain yield under both stress and non‐stress conditions (Mitra, 2001). Positive and highly significant correlations were observed between grain yield and the drought indices of the STI, MP, GMP, HM, YSI, and YI at both the Melkassa and Werer locations (Figure 2). Conversely, the remaining drought indices showed either no correlation or a low significant correlation with grain yield (Ys and Yi). For instance, the SSI displayed a nonsignificant correlation with grain yield under well‐watered conditions at Melkassa. Similarly, TOL exhibited either a nonsignificant or weak correlation with grain yield under stress conditions at Melkassa and Werer. Furthermore, the YR also showed a non‐significant correlation with grain yield under well‐watered conditions in Werer.

### 3.3 Clustering based on grain yield and drought indices

Cluster analysis was utilized to assess the variation among different genotypes and determine their drought tolerance levels based on significant indices associated with grain yield. The genotypes were grouped using the squared Euclidean distance to identify similarities and differences among them. The analysis included drought tolerance indices such as STI, MP, GMP, harmonic mean productivity (HMP), YSI, YI, and grain yield under stress and non‐stress conditions. The results revealed that the genotypes clustered into four main groups at both the Melkassa and Werer sites (Table 3; Figure S1; Table S3). Cluster IV (C‐IV) comprised 15 and 12 accessions (Werer and Melkassa, respectively) with high values for the STI, MP, GMP, YI, and HMP, indicating their strong tolerance to drought in the respective environments. Notably, accessions Acc. #28546 and Acc. #216739 in clusters C‐IV were identified as the most desirable for both growth conditions due to their high tolerance levels. On the other hand, cluster I (C‐I) consisted of 67 and 109 accessions in Melkassa and Werer, respectively, characterized by low grain yield values under water stress and all drought indices. These accessions in the cluster were classified as drought sensitive, highlighting their susceptibility to adverse conditions.

**TABLE 3:** Cluster summary based on drought indices and grain yield and water stress and well‐watered conditions at the Melkassa and Werer locations.

Melkassa											Werer									
Clusters	No of acc.		Ys	Yi	STI	MP	GMP	HMP	YSI	YI	No. of acc.	Ys	Yi	STI	MP	GMP	HM	YSI	YI	Decision
C‐I	67	Mean	585.67	1915.66	0.13	1250.67	1005.46	834.41	0.39	0.34	109	1317.69	2429.51	0.35	1873.60	1761.26	1661.99	0.58	0.68	sensitive
		Min	970.67	4496.00	0.01	2453.33	1790.86	1463.12	0.94	0.56		585.00	990.00	0.10	940.00	933.35	877.73	0.17	0.30	
		Max	197.33	501.33	0.36	394.67	344.82	301.26	0.08	0.11		1790.00	4870.00	0.67	2975.00	2475.50	2209.26	0.98	0.93	
C‐II	81	Mean	1627.13	2604.26	0.48	2115.69	2034.68	1960.42	0.65	0.93	30	2105.22	2611.11	0.60	2358.17	2340.06	2322.23	0.82	1.09	Semi‐sensitive
		Min	720.00	1338.67	0.14	1253.33	1134.20	1026.38	0.25	0.41		1890.00	2060.00	0.43	1980.00	1978.38	1976.77	0.62	0.98	
		Max	2533.33	4629.33	0.90	2962.67	2848.85	2797.32	0.99	1.45		2465.00	3190.00	0.82	2755.00	2739.69	2724.47	1.00	1.28	
C‐III	52	Mean	2614.63	4411.97	1.23	3513.30	3308.01	3137.59	0.65	1.50	64	2350.71	4063.68	1.04	3207.19	3062.30	2929.02	0.60	1.22	Moderate tolerant
		Min	1328.00	3194.67	0.94	2992.00	2905.19	2287.97	0.16	0.76		1485.00	2850.00	0.59	2475.00	2329.89	2193.29	0.26	0.77	
		Max	3797.33	8256.00	1.87	4792.00	4099.94	4087.92	0.99	2.18		3215.00	6245.00	1.77	4415.00	4017.88	3794.89	0.94	1.66	
C‐IV	15	Mean	4163.78	5947.11	2.51	5097.11	4988.04	4885.08	0.73	2.44	12	4223.64	4866.82	2.28	4545.23	4527.73	4510.37	0.89	2.19	Tolerant
		Min	3066.67	4592.00	2.03	4437.33	4266.58	4044.08	0.40	1.76		3610.00	3780.00	1.49	3695.00	3694.02	3693.04	0.76	1.87	
		Max	5341.33	8394.67	3.31	6925.33	6900.67	6876.09	0.96	3.64		5045.00	6250.00	3.45	5647.50	5615.27	5583.22	1.00	2.61	

Abbreviations: acc., accessions; GMP, geometric mean productivity; HMP, harmonic mean productivity; MP, mean productivity; STI, stress tolerance index; YI, yield index; Yi, mean yield under well‐watered conditions; Ys, mean yield under water stressed conditions; YSI, yield stability index.

### 3.4 Single nucleotide polymorphism summary and ML‐GWAS of drought indices

A comprehensive analysis of 22,010 SNP markers was conducted across 216 sorghum accessions. The dataset was filtered to exclude SNPs with an MAF < 0.05, resulting in a refined dataset of 17,637 SNPs (Figure S2). The genome‐wide marker density plot illustrated the distribution of markers from the study panel across the sorghum genome (Figure S2). The number of SNPs varied across chromosomes, ranging from 2682 (Sb‐01) to 1110 (Sb‐08), with marker densities varying from 33.3 Mb (Sb‐01) to 17.7 Mb (Sb‐08) across the genome. Transition mutations (51%) were more prevalent than transversion mutations (49%) (Figure S3), with an estimated 51.3% of total mutations involving A to G or vice versa (26%) and C to T or vice versa (25.3%). Transversion mutations accounted for 49.0% of the total mutations, with the highest frequency observed in C/G or G/C (17.8%) and the lowest in T/G and G/T (11.5%) among all 17,637 SNP markers.

**FIGURE 3:**
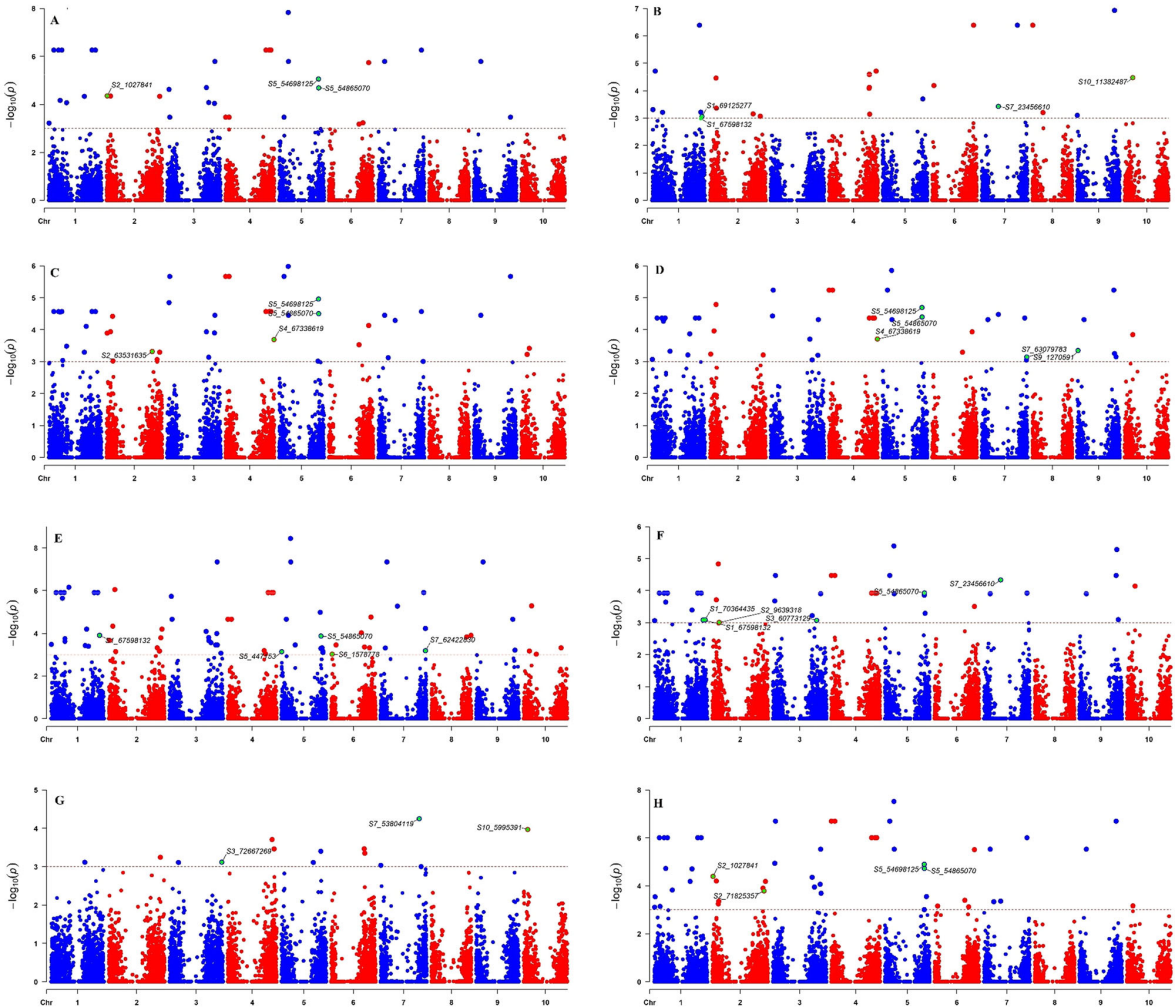
Significant quantitative trait nucleotides (QTNs) co‐detected simultaneously by using three or more multi‐locus genome‐wide association studies (ML‐GWAS) using grain yield traits in Melkassa experimental site: (A) Mean yield under water‐stressed condition (Ys), (B) mean yield under well‐watered condition (Yi), (C) harmonic mean (HM), (D) geometric mean productivity (GMP), (E) stress tolerance index (STI), (F) mean productivity (MP), (G) yield stability index (YSI), and (H) Harvest index (YI). The horizontal line indicates the significant threshold level. chr, chromosome.

**FIGURE 4:**
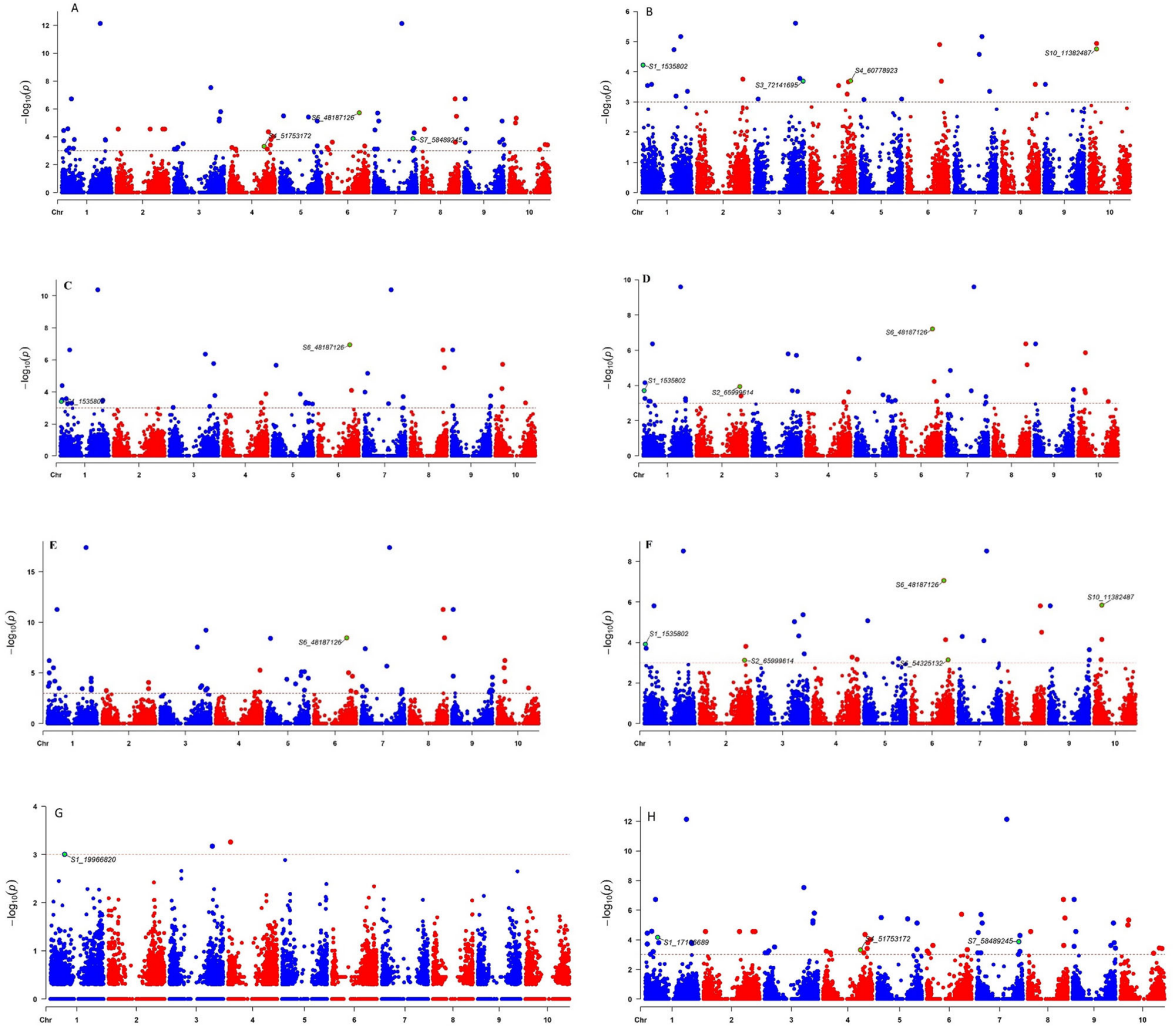
Significant quantitative trait nucleotides (QTNs) co‐detected simultaneously by using three or more multi‐locus genome‐wide association studies (ML‐GWAS) using grain yield traits in Werer experimental site: (A) Mean yield under water‐stressed condition (Ys), (B) mean yield under well‐watered condition (Yi), (C) harmonic mean (HM), (D) geometric mean productivity (GMP), (E) mean productivity (MP), (F) stress tolerance index (STI), (G) Harvest index (YI), and (H) yield stability index (YSI). The horizontal line indicates the significant threshold level. chr, chromosome.

A total of 32 QTNs were identified for drought indices and grain yield under stress and non‐stress conditions at Melkassa (Figure 3A–H; Table S4). Among these, S5_54865070 was detected among five drought indices (STI, MP, GMP, HM, and YI) and grain yield under water stress conditions. Among the three drought indices (GMP, HM, and YI), S5_54698125 QTN was associated with grain yield under stress conditions, followed by S1_67598132 (Yi and MP), S2_1027841 (Ys and Yi), S2_71825357 (Ys & Yi), S4_67338619 (GMP & HM), and S7_23456610 (Ys & MP). Moreover, a total of 22 QTNs were identified for drought indices and grain yield under water stress and non‐stress conditions at Werer (Figure 4A–H; Table S5). Among these, S1_1535802 QTN was detected in Yi, MP, GMP, and HM, followed by S6_48187126, which was identified in four traits, namely, Ys, STI, MP, GMP, and HM. Additionally, QTN S10_11382487 was detected in the Yi and MP populations in both the Melkassa and Werer environments.

### 3.5 Colocation with previously reported drought‐related QTLs

**FIGURE 5:**
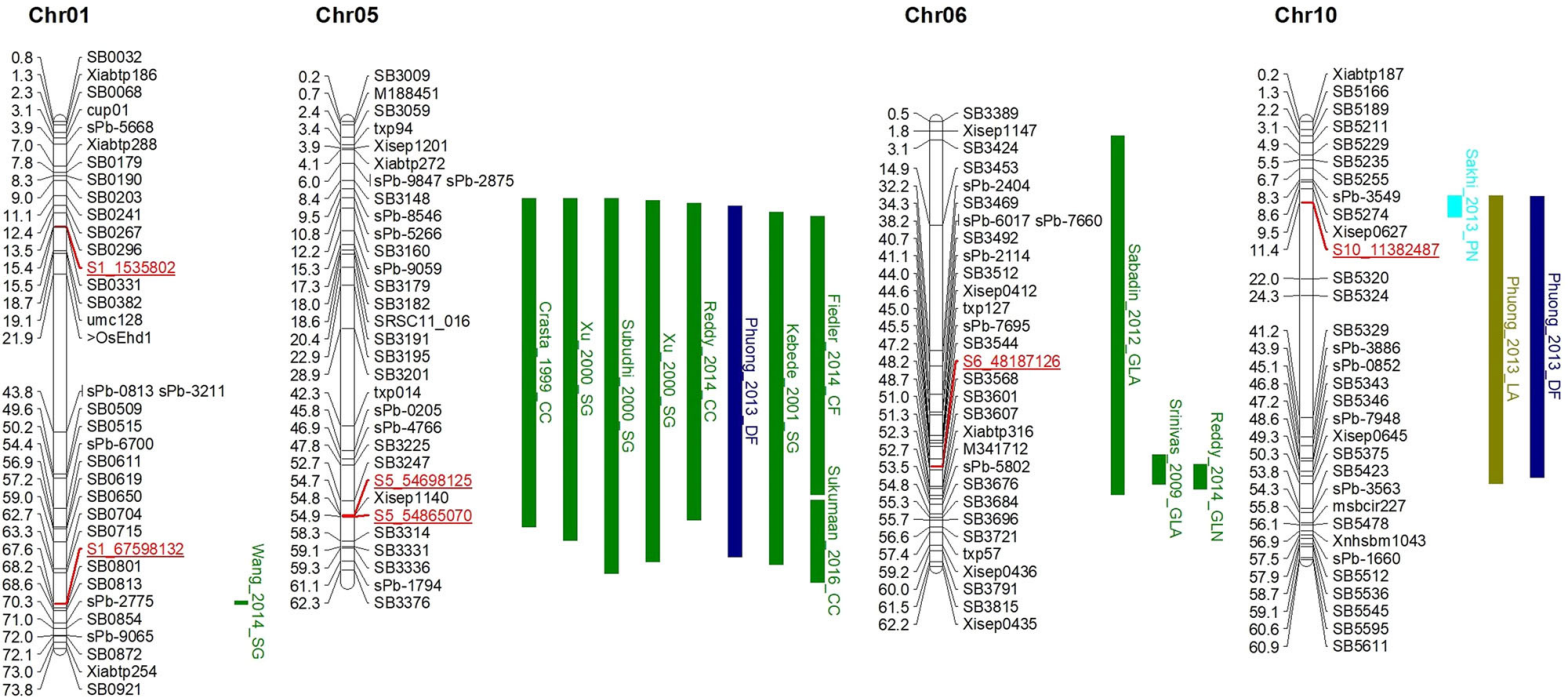
Significantly associated quantitative trait nucleotides (QTNs) at either two locations or at least three drought indices and colocated regions with previously studied quantitative trait loci (QTLs) on the consensus map. Green bar color represents previously studied stay‐green related QTLs, and other bar color represents other colocated QTLs from different studies. Red and underline marker indicates associated QTNs. The consensus markers are sourced from the Sorghumbase database v7. Physical distances are converted from base pairs (bp) to megabase pairs (Mbp) and using reference genome version V3. The QTL name indicates the first author, year of publication, and traits: CC, clorophyl content; DF, days to flowering; GLA, green leaf area; GLN, green leaf number; LA, leaf area; PN, panicle number; SG, stay‐green.

The identification of colocated QTNs with previously reported QTLs for drought adaptation was based on the significant association of QTNs detected in two locations or in at least three drought indices. A total of six QTNs were identified, either present in both locations (S10_11382487) or in at least three drought indices either at Melkassa or Werer (S1_1535802, S1_67598132, S5_54698125, S5_54865070, and S6_48187126) (Figure 5; Table S6). The S5_54865070 QTN was found in five drought indices and grain yield under stress conditions at Melkassa, while S6_48187126 was associated with four drought indices and grain yield under water‐stressed conditions at Werer. The S1_1535802 QTN was linked to three drought indices (MP, GMP, and HM) and grain yield under well‐watered conditions at Werer. Similarly, the S5_54698125 QTN was associated with three drought indices (GMP, HM, and YI) and grain yield under water stress conditions at Melkassa. The S1_67598132 QTN was linked to two drought indices (STI and MP) and grain yield under well‐watered conditions at Melkassa. Only the S10_11382487 QTN showed an association with drought indices (MPs) and grain yield under well‐watered conditions at both Werer and Melkassa. All these QTNs were colocalized with previously studied QTLs related to drought tolerance, except for S1_1535802. The majority of the colocated loci were reported for stay‐green‐related traits such as leaf senescence, chlorophyll content, green leaf area, and total number of green leaves. The S10_11382487 QTN colocated with panicle number, leaf area, and days to flowering (Figure 5; Table 6).

### 3.6 Candidate genes and annotation

The candidate genes identified in this study are associated with the LD decay regions surrounding six QTNs detected in two locations or in at least three drought indices (Tables S4 and S5). A total of 47 candidate genes were found upstream and downstream of the LD decay regions of these six QTNs (Table S7). Among these genes, 23 were linked to S1_1535802, 10 to S10_11382487, six to S1_67598132, six to S6_48187126, and three to S5_54698125. These genes were annotated with various protein functions. For instance, the *Sobic.001G018300* and *Sobic.001G018600* genes colocalized with S1_1535802, which is annotated as cytochrome P450. Additionally, *Sobic.001G018900*, *Sobic.001G017000*, and *Sobic.001G018800*, which are also colocalized with S1_1535802, were annotated as 40S, plasmid, and 60S ribosomal proteins (RPs), respectively. The *Sobic.001G345400* gene colocated with S1_67598132 was annotated as a putative ring‐H2 zinc finger protein, while *Sobic.001G345600* and *Sobic.001G345700*, also colocated with S1_67598132, were annotated as PLATZ transcription factors. The *Sobic.010G111400* gene colocated with QTN S10_11382487, which was identified in both locations, was annotated as a hypothetical protein homologous to MYB‐related transcription factor 81 in *Zea mays*. Furthermore, the *Sobic.005G110514* and *Sobic.005G110511* genes colocated with S5_54698125 were annotated as leucine‐rich repeat (LRR)‐containing proteins. The majority of the remaining colocalized genes were annotated as hypothetical proteins.

## 4 DISCUSSION

### 4.1 Plasticity of sorghum accessions for drought adaptation

The plasticity of sorghum genotypes in terms of drought adaptation is a crucial area of research, particularly when considering grain yield and various drought indices, such as the STI, MP, GMP, HMP, YSI, and YI (Paes de Camargo & Hubbard, 1999). This ability to maintain relatively high grain yield under water‐limited conditions makes them valuable for cultivation in drought‐prone regions. Understanding the plasticity of sorghum genotypes for drought adaptation is essential for breeding programs aimed at developing improved cultivars with enhanced drought tolerance. By identifying genotypes with high plasticity, breeders can incorporate these traits into new varieties, ultimately leading to more resilient and productive sorghum crops in drought‐prone areas.

In this study, significant variation in grain yield was observed among sorghum accessions under water stress and well‐watered conditions. Paes de Camargo and Hubbard (1999) also reported significant differences among sorghum varieties under water stress and non‐stress conditions. Similarly, drought indices also show variation among accessions. The variation in these grain yield and drought indices in sorghum is an important issue, especially in the context of climate change and the increasing frequency of drought events (Menezes et al., 2014). Because sorghum is cultivated in arid and semiarid regions, it is particularly susceptible to the adverse effects of drought (Ali et al., 2009; Jordan et al., 2012). Grain yield is a key indicator of the overall performance of sorghum under various environmental conditions, including drought stress (Paes de Camargo & Hubbard, 1999; Reddy et al., 2014). In this study, there was also a significant difference in yield reduction under water stress conditions. Hence, understanding the relationship between grain yield and drought indices variation in sorghum is crucial for developing strategies to mitigate the impact of water scarcity on crop productivity.

In this study, four varieties, Kem‐Kem, Melkam, Wedi‐Aker, and B‐35, were included. Among these, Melkam and B‐35 performed well (among the top 10 accessions) in the Melkassa and Werer environments, respectively. The improved sorghum variety, Melkam, has been newly introduced in the last decade. Hence, the yield of four accessions (Acc. #220268, Acc. #234115, Acc. #216739, and Acc. #216736) was greater than that of the Melkam variety under stress conditions in Melkassa. However, the B‐35 variety had the greatest yield (5045.0 kg/ha) compared to all the other accessions as well as varieties under water stress conditions in Werer. This indicates that these two varieties may have different genetic backgrounds that contribute to their drought resistance. These genetic differences can result in variations in how well a variety performs under drought conditions in different locations. Similarly, F. Girma et al. (2020) reported less yield reduction in Melkam and B‐35 varieties than in other genotypes under water stress and non‐stress conditions with a high leaf green area at the maturity stage.

In addition to grain yield, drought indices such as the STI, MP, GMP, HMP, YSI, and YI offer valuable tools for evaluating the performance of sorghum genotypes under water‐limited conditions (Paes de Camargo & Hubbard, 1999; Xin et al., 2022). These indices provide insights into the genotypic response to drought stress, considering both yield stability and productivity (Choudhary et al., 2021). Sorghum genotypes with high plasticity for drought adaptation demonstrate consistent performance across these indices, indicating their ability to maintain grain yield and physiological functions under challenging environmental conditions. A suitable index must have a highly significant correlation with grain yield under both stress and non‐stress conditions (Mitra, 2001). Correlation analysis revealed that grain yield under water stress conditions had a positive and highly significant correlation with grain yield under non‐stress conditions at both the Melkassa and Werer sites (Figure 2). Similarly, Ashraf et al. (2015) reported a positive and highly significant correlation with grain yield under stress and non‐stress conditions in wheat (*Triticum aestivum*) genotypes. This result indicated that high grain yield under optimal conditions necessarily results in improved yield under stress conditions.

At both locations, positive and highly significant correlations were estimated between grain yield under both stress and non‐stress conditions and drought indices such as the STI, MP, GMP, HMP, YSI, and YI (Figure 2). These findings indicate that these criteria can be used to distinguish drought‐tolerant genotypes with high grain yields under stressful and non‐stressed environments (Mitra, 2001). The most appropriate indices for the selection of drought‐tolerant cultivars are indices showing a relatively high correlation with grain yield under both environmental conditions (Farshadfar & Sutka, 2003; Khalilzade & Karbalai‐Khiavi, 2002; Wadan et al., 2015). Similarly, Menezes et al. (2014) reported positive and significant correlations of yield under non‐stress (Yp) and stress (Ys) with the GMP, MP, HMP, and STI, indicating that these factors are better predictors of Yp and Ys than other tolerance indices, such as the SSI, YSI, and YI.

### 4.2 Cluster analysis identified tolerant accessions

The accessions were grouped into four major clusters at both the Melkassa and Werer locations. Among these, the fourth cluster (C‐IV) consisted of 15 and 12 accessions from the Melkassa and Werer locations, respectively. This cluster consisted of accessions that performed well under well‐watered and water‐stressed environments and had higher values for indices and high values for the STI, MP, GMP, YI, and HMP; thus, they are considered to be the most desirable (tolerant) genotype. In particular, the Acc. #28546 and Acc. #216739 accessions were included in the fourth cluster C‐IV in both the experimental Melkassa and Werer locations. Hence, they are considered to be the most desirable genotypes for both growth conditions (tolerant group) due to these two varieties might have a different interaction with the environment. However, nearly 42 accessions belonged to the first cluster (C‐I) in both locations with the lowest drought induction and grain yield values; this cluster consists of accessions sensitive to drought. Similarly, Abraha et al. (2015) grouped sorghum accessions into five clusters based on drought induction: one group (C‐IV) with the highest drought indices values (MP, GMP, and STI) which was considered to be the most desirable cluster, while the other two clusters with lower drought indices values were considered susceptible to drought. Moreover, Choudhary et al. (2021) identified tolerant sorghum genotypes based on yield performance under well‐watered and water‐stressed environments and drought indices.

### 4.3 Multi‐locus genome‐wide association studies

ML‐GWAS have emerged as a promising approach for identifying genomic regions associated with complex traits (Rakitsch et al., 2013). Traditional single‐locus GWAS models, often employing ordinary mixed models, may not fully capture the influence of loci with large effects. ML‐GWAS models offer a more efficient and reliable framework for mapping genomic regions by simultaneously estimating the effects of all markers (Zhong et al., 2021). This simultaneous estimation can lead to a more comprehensive understanding of the genetic architecture of complex traits. Hence, ML‐GWAS for drought indices such as the STI, MP, GMP, HMP, YSI, and YI can provide valuable insights into the genetic basis of sorghum drought tolerance. By examining the genetic variations associated with these drought indices, we identified candidate genes and genomic regions that contribute to sorghum's ability to withstand and adapt to drought stress. A total of 32 QTNs were identified for drought indices and grain yield stress and non‐stress conditions in Melkassa (Figure 3), while a total of 22 QTNs were identified for drought indices and grain yield under water stress and non‐stress conditions in Werer (Figure 4). Among these, only one QTN (S10_11382487) was detected in terms of grain yield and MP index in both the Melkassa and Werer environments. Additionally, S1_1535802, S1_67598132, S5_54698125, S5_54865070, and S6_48187126 were significant QTNs associated with at least three drought indices in both the Melkassa and Werer locations. Xin et al. (2022) identified a total of 79 significant markers associated with drought induces in sorghum, including 19 for MP, 19 for the resistance drought index and 41 for the STI. The detection of different QTLs in different locations within the same sorghum population reflects the genetic complexity and environmental interactions that influence trait expression in plants. Similarly, Ballesta et al. (2019) used drought indices in wheat to identify 45 SNPs associated with more than one tolerance index for different grain yield‐related traits.

The different QTNs at different locations might indicate that genotypes respond to different mechanisms or different regions at different locations. This is in line with the performance of the varieties, that is, the Melkam variety performed well in Melkassa, while the B‐35 variety performed well in Werer. Different regions of the genome may contain different sets of genes that influence specific traits. This genetic diversity can lead to the detection of different QTLs in different locations. Another reason could be population structure. The accessions were collected from different backgrounds for post‐flowering drought tolerance. These populations can exhibit genetic heterogeneity due to factors such as breeding history, selection pressure, and gene flow. This population structure can lead to the detection of different QTLs in different locations within the same population.

### 4.4 The majority of QTNs colocalized with priori QTLs related to stay‐green

The colocation focused on six QTNs detected either in both locations (S10_11382487) or in at least three drought induces in either the Melkassa or Werer location (S1_1535802, S1_67598132, S5_54698125, S5_54865070, and S6_48187126). All these QTNs colocalized with QTLs reported for stay‐green traits such as green area (Kebede et al., 2001; Subudhi et al., 2000; H. Wang et al., 2014; Xu et al., 2000), chlorophyll content (Crasta et al., 1999; Reddy et al., 2014; Sukumaran et al., 2016), chlorophyll fluorescence (Fiedler et al., 2014), green leaf area (Sabadin et al., 2012; Srinivas et al., 2009), and total number of green leaves (Reddy et al., 2014). Staying green is a physiological trait that refers to the delayed senescence of leaves during periods of drought stress, allowing plants to maintain green and functional leaves for a longer duration (A. Borrell et al., 2001). This trait has been recognized as a crucial mechanism for post‐flowering drought tolerance in sorghum and other crops (A. Borrell et al., 1999; A. K. Borrell, Mullet et al., 2014; Kamal et al., 2018). The ability of plants to retain green leaves under drought conditions is associated with enhanced photosynthetic capacity, improved water use efficiency, and improved recovery after drought stress is alleviated. Compared with their non‐stay‐green counterparts, stay‐green sorghum varieties have been shown to exhibit improved post‐flowering drought recovery (Burke et al., 2010; Harris et al., 2007). These varieties are able to maintain relatively high levels of chlorophyll and photosynthetic activity during drought stress, which contributes to improved yield stability and productivity under challenging environmental conditions (A. K. Borrell et al., 2014). The stay‐green trait in sorghum is controlled by multiple genes and regulatory pathways involved in chlorophyll degradation, nutrient remobilization, and stress signaling (Hörtensteiner, 2009; Thomas & Ougham, 2014).

### 4.5 Candidate genes contributing to stay‐green traits

*Sobic.001G018300* and *Sobic.001G018600* are colocated genes with S1_1535802 QTN. These genes were annotated similarly to the *cytochrome P450* (*CYP*) protein. Cytochrome P450s are among the largest protein‐coding gene families involved in plant metabolism, including hormone biosynthesis and catabolism, as well as the synthesis of primary and secondary metabolites (Mizutani & Ohta, 2010; Tamiru et al., 2015). The *dwarf and small seed 1* (*dss1*) mutant gene in rice (*Oryza sativa*) corresponds to the P450 gene (*CYP96B4/SD37*), which is characterized by the accumulation of abscisic acid (ABA) and metabolites, reduced gebberellic acid (GA) levels, and enhanced tolerance to drought stress compared to those in wild plants (Tamiru et al., 2015). Similarly, the *CYP707A* (*ABA8Ox*) gene was found to be upregulated when maize was exposed to water deficit conditions (Y. Li & Wei, 2020). *Cytochrome P450* has also been found to play a role in the synthesis of leaf lignin and grain formation in plants exposed to drought stress (Pandian et al., 2020). For example, *CYP96A8* is speculated to be involved in lignin biosynthesis and drought response‐related functions (Hu et al., 2009). Additionally, *cytochrome P450* (*CYP89A9*) is involved in the formation of major chlorophyll catabolites during leaf senescence in Arabidopsis [*Arabidopsis thaliana* (L.) Heynh] (Christ et al., 2013). Similarly, *cytochrome P450* (*Zm00001eb384100*), which is highly significant for maize (*Z. mays*) greening, is related to leaf senescence and mainly participates in jasmonic acid reactions (Zheng et al., 2023).

*Sobic.001G018900*, *Sobic.001G017000*, and *Sobic.001G018800* are also colocalized with S1_1535802 QTN annotated with 40S plasmid and 60S RP, respectively. Shiraku et al. (2021) reported that the knockdown of 60S RP (L14‐2) has potential regulatory roles in enhancing drought tolerance in cotton (*Gossypium hirsutum* L.). Similarly, Moin et al. (2017) reported the upregulation of RP genes in both shoot and root tissues under drought conditions in rice. One of the 60S RPs that has been associated with the stay‐green trait in plants is RPL23. RPL23 is a component of the large subunit of the ribosome and plays a crucial role in protein synthesis. Studies have shown that RPL23 is involved in regulating leaf senescence and stress responses in plants, including the maintenance of chlorophyll levels and photosynthetic activity under drought conditions (Lencina et al., 2019; Schippers & Mueller‐Roeber, 2010).

Similar to the putative ring H2 zinc finger protein, the *Sobic.001G345400* gene colocated with S1_67598132 QTN was annotated. This protein plays important roles in the regulation of abiotic stress tolerance, suggesting that it is linked to ABA signaling in plants (Y. Liu et al., 2024; Zeng et al., 2014). RING zinc finger proteins usually act as E3 ubiquitin ligases in the abiotic stress response through ABA, mitogen‐activated protein kinase, and ethylene signaling pathways (Han et al., 2022). RING‐H2 contains protein response domains to drought in wheat (Kam et al., 2007); similarly, the RING‐H2 finger protein regulates growth and responses to abiotic stress in rice (H. Liu et al., 2008). The mutant RING zinc finger protein (atairp3) exhibited impaired stomatal closure and increased sensitivity to drought in *A. thaliana* (J. H. Kim & Kim, 2013). Transgenic hybrid poplars overexpressing the RING protein family (*PtXERICO*) exhibit enhanced drought tolerance with reduced transpirational water loss and ion leakage (M.‐H. Kim et al., 2020). Furthermore, *T. aestivum* transgenics plants survive longer when subjected to drought stress for 14 days than do wild‐type plants and exhibit increased fresh weight, improved growth, increased chlorophyll accumulation, increased membrane stability, and increased proline content (Agarwal & Khurana, 2020).

The *Sobic.001G345600* and *Sobic.001G345700* genes colocalized with the S1_67598132 QTN gene, which is annotated as a PLATZ transcription factor (PLATZ). Plant AT‐rich sequence and zinc binding (PLATZ) transcription factors are a class of plant‐specific zinc‐dependent DNA‐binding proteins that function in abiotic stress responses and plant development (S. Liu et al., 2020; Zhao et al., 2022). Zhao et al. (2022) reported that PLATZ (GmPLATZ17) inhibits drought tolerance by interacting with *GmDREB5* in soybean (*Glycine max*).

The *Sobic.010G111400* gene colocalized with QTN, which was identified in both locations. This gene was annotated as a hypothetical protein that is homologous to the MYB‐related transcription factor 81 of *Z. mays*. These MYB transcription factors can directly or indirectly regulate the expression of multiple stress‐related genes in plants to address adverse external environments. Some MYB transcription factors are involved in the drought response through their regulation of lateral root growth (Shin et al., 2007), stomata (Cominelli et al., 2010), and recovery in drought‐treated flowers (Mandaokar & Browse, 2009). Additionally, co‐expression of members of the MYB transcription factors with stay‐green (*SlSGR*) genes has been reported in tomato (*Solanum lycopersicum*) (Uluisik et al., 2022). Moreover, the MYB TF, AtMYB60, is involved in stomatal conductance regulation to control water loss in plants (Oh et al., 2011). The mutant (*myb60*) enhances plant drought tolerance via reduced water loss (Cominelli et al., 2005). The overexpression of cotton MYB transcription factors (*GaMYB85*) can promote the accumulation of free proline and chlorophyll in transgenic plants (Butt et al., 2017). MYB transcription factors participate in drought tolerance related to leaf permeability by regulating the synthesis of flavonoids and cuticles to reduce the water loss rate (X. Wang et al., 2021).

The *Sobic.005G110514* and *Sobic.005G110511* genes colocalized with the S5_54698125 QTN gene and were annotated as LRR‐containing proteins. Various LRRs are known for their roles in tolerance and adaptive mechanisms during drought stress. LRR (LP2) directly regulates *drought‐related transcription factors* and interacts with *drought‐responsive aquaporin* proteins by functioning as a negative regulator of drought stress (Tyagi & Upadhyay, 2023). Wu et al. (2015) reported that the overexpression of this gene in rice lowered H_2_O_2_ levels and restricted stomatal closure in leaves. Early and late upregulation of these genes has been reported in *T. aestivum* under drought stress treatment (Shumayla et al., 2016). Overexpression of the LRR family (*PdERECTA*) in transgenic Arabidopsis resulted in enhanced long‐term water use efficiency through improvements in the photosynthetic rate and decreased transpiration rate and stomatal density (Xing et al., 2011). ERECTA (ER) is a LRR‐receptor‐like kinase gene from sorghum (SbER) expressed in Arabidopsis and maize that confers increased drought tolerance, especially in regard to water‐use efficiency, increasing the net photosynthetic rate in maize under drought stress (H. Li et al., 2019). The Arabidopsis ER genes improve transpiration efficiency by influencing the development of epidermal cells and mesophyll cells, stomatal density, and leaf porosity (Shpak, 2013). In addition, the ER balances transpiration and photosynthesis by improving leaf characteristics and the photosynthetic capacity of mesophyll cells (Masle et al., 2005).

## 5 CONCLUSION

The plasticity of sorghum genotypes for drought adaptation, particularly in terms of grain yield and drought indices, is a critical area of research with significant implications for agricultural productivity and food security. By identifying and understanding the genetic basis of plasticity in sorghum, researchers, breeders, and farmers can work together to develop and adopt improved sorghum varieties that are better equipped to thrive in water‐limited conditions. This will ultimately contribute to sustainable agriculture and the resilience of farming communities in drought‐prone regions. The results from this study are very useful for planning future sorghum breeding programs, particularly in water‐stressed areas. Significant genetic variation in grain yield was observed among the accessions under both water stress and well‐watered regimes. Selection based on a combination of indices may provide a more suitable criterion for improving the drought tolerance of sorghum. In this evaluation of drought stress indices, the STI, MP, GMP, HMP, YSI, and YI were identified as appropriate indices for selecting drought‐tolerant sorghum accessions. Selection by these indices can be useful for identifying a genotype with desirable yield under both post‐flowering stress and non‐stress conditions. Moreover, it is recommended that the selection be performed based on the use of several indices instead of only one index. Cluster analysis revealed that the genotypes whose grain yield was significantly associated with both water stress and well water tended to be grouped into four groups: tolerant, moderately tolerant, semi‐sensitive, and sensitive accession groups. Multiple statistical procedures used in this study revealed that among all the accessions, Acc. #28546 and Acc. #216739 were the most drought tolerant in both environments.

The colocated genes with specific QTNs identified in this study play crucial roles in plant responses to abiotic stress, particularly maintaining a stay‐green color. Genes such as *Sobic.001G018300* and *Sobic.001G018600*, annotated as *cytochrome P450* proteins, are involved in hormone biosynthesis, metabolite accumulation, and chlorophyll content. Similarly, the presence of RPs near the QTNs suggests potential regulatory roles in enhancing drought tolerance. The putative ring‐H2 zinc finger protein associated with the gene *Sobic.001G345400* is linked to ABA signaling and abiotic stress tolerance mechanisms. Additionally, PLATZ transcription factors found near certain QTNs are implicated in abiotic stress responses and plant development. The hypothetical protein encoded by *Sobic.010G111400* shares homology with MYB‐related transcription factors known for regulating stress‐related genes in response to external challenges. Finally, the LRR‐containing proteins encoded by *Sobic.005G110514* and *Sobic.005G110511* have been associated with drought tolerance mechanisms through interactions with drought‐responsive proteins. The identification of these genes and their functions provides valuable insights into the molecular mechanisms underlying drought stress responses in plants. Further research on these genes may lead to the development of improved drought‐tolerant crop varieties.

## AUTHOR CONTRIBUTIONS

**Yirgalem Tsehaye**: Conceptualization; data curation; formal analysis; writing—original draft; writing—review and editing. **Temesgen M. Menamo**: Conceptualization; data curation; formal analysis; methodology; supervision; writing—original draft; writing—review and editing. **Fetien Abay**: Conceptualization; supervision; writing—review and editing. **Taye Tadesse**: Conceptualization; resources; supervision; validation; writing—original draft; writing—review and editing. **Kassahun Bantte**: Conceptualization; supervision; validation; writing—original draft; writing—review and editing.

## CONFLICT OF INTEREST STATEMENT

The authors declare no conflicts of interest.

## Supporting information

Supplementary Figure S1: Dendrogram of 216 sorghum accessions based on drought induces at Melkassa and Werer experimental site.

Supplementary Figure S2: SNP makers and marker density (Mb) across 10 sorghum chromosomes.

Supplementary Figure S3: Transition and transversion based on bi‐allelic SNP markers. Tv: Transversions; Ts: Transitions; A: Adenine; T: Thymine; G: Guanine; C: Cytosine. The first combination nucleotide refers the mutation in individual accession and the 2nd nucleotide combination refers the reference genome nucleotide.

Supplementary Table S1: List of genetic materials with source of origins and planting group based on maturity.

Supplementary Table S2: Estimation of drought tolerance indices based on total root yield of sorghum genotypes under water stress and non‐stress conditions in Melkassa.

Supplementary Table S3: List of accessions per cluster in Melkassa.

Supplementary Table S4: List of significant QTNs codetected simultaneously by using three or more multilocus GWAS methods for drought indices and using grain yield traits in Melkassa.

**Supplementary Table 5**: List of significant QTNs co‐detected simultaneously by using three or more multi‐locus GWAS methods for drought indices and using grain yield traits in Werer.

**Supplementary Table S6**: List of significantly associated QTNs at either two locations or at least three drought‐induced and colocated regions with previously studied QTLs.

**Supplementary Table S7**: Candidate genes with significant QTNs identified for drought adaptation in this study.

## Data Availability

All data are publicly accessible. A genotypic list with geographical information, SNP genotype, and phenotypic data is available from the digital repository (https://doi.org/10.5061/dryad.c59zw3rhk) and provided in the online resource.

## References

[tpg220505-bib-0001] Abraha, T. , Nyende, A. B. , Githiri, S. M. , Kasili, R. W. , & Araia, W. (2015). Identification of sorghum (*Sorghum bicolor* L. Moench) landraces tolerant to post flowering drought stress using drought tolerance indices. Journal of Plant Breeding and Crop Science, 7(7), 211–218.

[tpg220505-bib-0002] Agarwal, P. , & Khurana, P. (2020). TaZnF, a C3HC4 type RING zinc finger protein from *Triticum aestivum* is involved in dehydration and salinity stress. Journal of Plant Biochemistry and Biotechnology, 29, 395–406. 10.1007/s13562-019-00546-8

[tpg220505-bib-0003] Ali, M. A. , Abbas, A. , Niaz, S. , Zulkiffal, M. , & Ali, S. (2009). Morpho‐physiological criteria for drought tolerance in sorghum (*Sorghum bicolor*) at seedling and post‐anthesis stages. International Journal of Agriculture and Biology, 11(6), 674–680.

[tpg220505-bib-0004] Armero, C. , Cabras, S. , Castellanos, M. E. , & Quirós, A. (2019). Two‐stage Bayesian approach for GWAS with known genealogy. Journal of Computational and Graphical Statistics, 28(1), 197–204. 10.1080/10618600.2018.1483828

[tpg220505-bib-0005] Ashraf, A. E.‐M. , Abd El‐Shafi, M. , Gheith, E. M. S. , & Suleiman, S. H. (2015). Using different statistical procedures for evaluating drought tolerance indices of bread wheat genotypes. Advances in Agriculture and Biology, 4(1), 19–30.

[tpg220505-bib-0006] Assefa, A. , Bezabih, A. , Girmay, G. , Alemayehu, T. , & Lakew, A. (2020). Evaluation of sorghum (*Sorghum bicolor* (L.) Moench) variety performance in the lowlands area of Wag Lasta, north eastern Ethiopia. Cogent Food & Agriculture, 6(1), Article 1778603.

[tpg220505-bib-0007] Ballesta, P. , Mora , F. , & Del Pozo, A. (2019). Association mapping of drought tolerance indices in wheat: QTL‐rich regions on chromosome 4A. Scientia Agricola, 77, e20180153.

[tpg220505-bib-0008] Barilli, E. , Cobos, M. J. , Carrillo, E. , Kilian, A. , Carling, J. , & Rubiales, D. (2018). A high‐density integrated DArTseq SNP‐based genetic map of *Pisum fulvum* and identification of QTLs controlling rust resistance. Frontiers in Plant Science, 9, Article 167. 10.3389/fpls.2018.00167 29497430 PMC5818415

[tpg220505-bib-0009] Bernardo, R. (2008). Molecular markers and selection for complex traits in plants: Learning from the last 20 years. Crop Science, 48(5), 1649–1664. 10.2135/cropsci2008.03.0131

[tpg220505-bib-0010] Borrell, A. , Bidinger, F. R. , & Sunitha, K. (1999). Stay‐green trait associated with yield in recombinant inbred sorghum lines varying in rate of leaf senescence. International Sorghum and Millets Newsletter, 40, 31–34.

[tpg220505-bib-0011] Borrell, A. , Hammer, G. , & Van Oosterom, E. (2001). Stay‐green: A consequence of the balance between supply and demand for nitrogen during grain filling? Annals of Applied Biology, 138(1), 91–95. 10.1111/j.1744-7348.2001.tb00088.x

[tpg220505-bib-0012] Borrell, A. K. , Mullet, J. E. , George‐Jaeggli, B. , Van Oosterom, E. J. , Hammer, G. L. , Klein, P. E. , & Jordan, D. R. (2014). Drought adaptation of stay‐green sorghum is associated with canopy development, leaf anatomy, root growth, and water uptake. Journal of Experimental Botany, 65(21), 6251–6263. 10.1093/jxb/eru232 25381433 PMC4223986

[tpg220505-bib-0014] Bouslama, M. , & Schapaugh, W. T., Jr. (1984). Stress tolerance in soybeans. I. Evaluation of three screening techniques for heat and drought tolerance. Crop Science, 24(5), 933–937. 10.2135/cropsci1984.0011183X002400050026x

[tpg220505-bib-0015] Bradbury, P. J. , Zhang, Z. , Kroon, D. E. , Casstevens, T. M. , Ramdoss, Y. , & Buckler, E. S. (2007). TASSEL: Software for association mapping of complex traits in diverse samples. Bioinformatics, 23(19), 2633–2635. 10.1093/bioinformatics/btm308 17586829

[tpg220505-bib-0016] Browning, B. L. , Zhou, Y. , & Browning, S. R. (2018). A one‐penny imputed genome from next‐generation reference panels. The American Journal of Human Genetics, 103(3), 338–348. 10.1016/j.ajhg.2018.07.015 30100085 PMC6128308

[tpg220505-bib-0017] Burke, J. J. , Franks, C. D. , Burow, G. , & Xin, Z. (2010). Selection system for the stay‐green drought tolerance trait in sorghum germplasm. Agronomy Journal, 102(4), 1118–1122. 10.2134/agronj2009.0465

[tpg220505-bib-0018] Butt, H. I. , Yang, Z. , Gong, Q. , Chen, E. , Wang, X. , Zhao, G. , Ge, X. , Zhang, X. , & Li, F. (2017). *GaMYB85*, an R2R3 MYB gene, in transgenic *Arabidopsis* plays an important role in drought tolerance. BMC Plant Biology, 17(1), Article 142. 10.1186/s12870-017-1078-3 28830364 PMC5568319

[tpg220505-bib-0019] Choudhary, R. , Biradar, D. P. , & Katageri, I. S. (2021). Evaluation of sorghum RILs for moisture stress tolerance using drought tolerance indices. The Pharma Innovation Journal, 10(4), 39–45.

[tpg220505-bib-0020] Christ, B. , Süssenbacher, I. , Moser, S. , Bichsel, N. , Egert, A. , Müller, T. , Kräutler, B. , & Hörtensteiner, S. (2013). Cytochrome P450 CYP89A9 is involved in the formation of major chlorophyll catabolites during leaf senescence in *Arabidopsis* . The Plant Cell, 25(5), 1868–1880. 10.1105/tpc.113.112151 23723324 PMC3694711

[tpg220505-bib-0021] Cominelli, E. , Galbiati, M. , & Tonelli, C. (2010). Transcription factors controlling stomatal movements and drought tolerance. Transcription, 1(1), 41–45. 10.4161/trns.1.1.12064 21327157 PMC3035188

[tpg220505-bib-0022] Cominelli, E. , Galbiati, M. , Vavasseur, A. , Conti, L. , Sala, T. , Vuylsteke, M. , Leonhardt, N. , Dellaporta, S. L. , & Tonelli, C. (2005). A guard‐cell‐specific MYB transcription factor regulates stomatal movements and plant drought tolerance. Current Biology, 15(13), 1196–1200. 10.1016/j.cub.2005.05.048 16005291

[tpg220505-bib-0023] Crasta, O. R. , Xu, W. W. , Rosenow, D. T. , Mullet, J. , & Nguyen, H. T. (1999). Mapping of post‐flowering drought resistance traits in grain sorghum: Association between QTLs influencing premature senescence and maturity. Molecular and General Genetics, 262(3), 579–588. 10.1007/s004380051120 10589847

[tpg220505-bib-0024] Elias Duresso, M. , Lule, D. , Tirfessa, A. , Gelmesa, D. , Tesso, T. , Menamo, T. , & Serba, D. D. (2023). Genetic diversity in Ethiopian sorghum germplasm for root system architecture and trait association. Rhizosphere, 27, 100759. 10.1016/j.rhisph.2023.100759 PMC12807215 38361379

[tpg220505-bib-0025] Elshire, R. J. , Glaubitz, J. C. , Sun, Q. , Poland, J. A. , Kawamoto, K. , Buckler, E. S. , & Mitchell, S. E. (2011). A robust, simple genotyping‐by‐sequencing (GBS) approach for high diversity species. PLoS ONE, 6(5), e19379. 10.1371/journal.pone.0019379 21573248 PMC3087801

[tpg220505-bib-0026] Enyew, M. , Feyissa, T. , Carlsson, A. S. , Tesfaye, K. , Hammenhag, C. , Seyoum, A. , & Geleta, M. (2022). Genome‐wide analyses using multi‐locus models revealed marker‐trait associations for major agronomic traits in *Sorghum bicolor* . Frontiers in Plant Science, 13, 999692. 36275578 10.3389/fpls.2022.999692 PMC9585286

[tpg220505-bib-0027] FAOSTAT . (2022). Agriculture organization of the United Nations . FAO. http://faostat3.fao.org/faostat‐gateway/go/to/download/Q/QC/S

[tpg220505-bib-0114] Farshadfar, E. , & Sutka, J. (2003). Multivariate analysis of drought tolerance in wheat substitution lines. Cereal Research Communications, 31(1–2), 33–40. 10.1007/bf03543247

[tpg220505-bib-0028] Fernandez, G. C. J. (1992). Effective selection criteria for assessing plant stress tolerance. In C. G. Kuo (Ed.), Proceedings of the international symposium on adaptation of vegetables and other food crops in temperature and water stress (pp. 257–270). AVRDC Publication.

[tpg220505-bib-0029] Fiedler, K. , Bekele, W. A. , Duensing, R. , Gründig, S. , Snowdon, R. , Stützel, H. , Zacharias, A. , & Uptmoor, R. (2014). Genetic dissection of temperature‐dependent sorghum growth during juvenile development. Theoretical and Applied Genetics, 127(9), 1935–1948. 10.1007/s00122-014-2350-7 25023408

[tpg220505-bib-0030] Fischer, R. , & Maurer, R. (1978). Drought resistance in spring wheat cultivars. I. Grain yield responses. Australian Journal of Agricultural Research, 29(5), 897–912. 10.1071/AR9780897

[tpg220505-bib-0031] Gavuzzi, P. , Rizza, F. , Palumbo, M. , Campanile, R. G. , Ricciardi, G. L. , & Borghi, B. (1997). Evaluation of field and laboratory predictors of drought and heat tolerance in winter cereals. Canadian Journal of Plant Science, 77(4), 523–531. 10.4141/P96-130

[tpg220505-bib-0032] Girma, F. , Mekbib, F. , Mindaye, T. T. , Menamo, T. M. , & Bantte, K. (2020). Phenotyping sorghum [*Sorghum bicolor* (L.) Moench] for drought tolerance with special emphasis to root angle. African Journal of Agricultural Research, 16(8), 1213–1222.

[tpg220505-bib-0033] Girma, G. , Nida, H. , Seyoum, A. , Mekonen, M. , Nega, A. , Lule, D. , Dessalegn, K. , Bekele, A. , Gebreyohannes, A. , Adeyanju, A. , Tirfessa, A. , Ayana, G. , Taddese, T. , Mekbib, F. , Belete, K. , Tesso, T. , Ejeta, G. , & Mengiste, T. (2019). A large‐scale genome‐wide association analyses of Ethiopian sorghum landrace collection reveal loci associated with important traits. Frontiers in Plant Science, 10, Article 691. 10.3389/fpls.2019.00691 31191590 PMC6549537

[tpg220505-bib-0034] Gladman, N. , Olson, A. , Wei, S. , Chougule, K. , Lu, Z. , Tello‐Ruiz, M. , Meijs, I. , Van Buren, P. , Jiao, Y. , Wang, B. , Kumar, V. , Kumari, S. , Zhang, L. , Burke, J. , Chen, J. , Burow, G. , Hayes, C. , Emendack, Y. , Xin, Z. , & Ware, D. (2022). SorghumBase: A web‐based portal for sorghum genetic information and community advancement. Planta, 255(2), Article 35. 10.1007/s00425-022-03821-6 35015132 PMC8752523

[tpg220505-bib-0035] Golestani Araghi, S. , & Assad, M. T. (1998). Evaluation of four screening techniques for drought resistance and their relationship to yield reduction ratio in wheat. Euphytica, 103, 293–299. 10.1023/A:1018307111569

[tpg220505-bib-0036] Goodstein, D. M. , Shu, S. , Howson, R. , Neupane, R. , Hayes, R. D. , Fazo, J. , Mitros, T. , Dirks, W. , Hellsten, U. , Putnam, N. , & Rokhsar, D. S. (2012). Phytozome: A comparative platform for green plant genomics. Nucleic Acids Research, 40(D1), D1178–D1186. 10.1093/nar/gkr944 22110026 PMC3245001

[tpg220505-bib-0037] Hadebe, S. T. , Modi, A. T. , & Mabhaudhi, T. (2017). Drought tolerance and water use of cereal crops: A focus on sorghum as a food security crop in sub‐Saharan Africa. Journal of Agronomy and Crop Science, 203(3), 177–191. 10.1111/jac.12191

[tpg220505-bib-0038] Han, G. , Qiao, Z. , Li, Y. , Yang, Z. , Wang, C. , Zhang, Y. , Liu, L. , & Wang, B. (2022). RING zinc finger proteins in plant abiotic stress tolerance. Frontiers in Plant Science, 13, Article 877011. 10.3389/fpls.2022.877011 35498666 PMC9047180

[tpg220505-bib-0039] Harris, K. , Subudhi, P. , Borrell, A. , Jordan, D. , Rosenow, D. , Nguyen, H. , Klein, P. , Klein, R. , & Mullet, J. (2007). Sorghum stay‐green QTL individually reduce post‐flowering drought‐induced leaf senescence. Journal of Experimental Botany, 58(2), 327–338. 10.1093/jxb/erl225 17175550

[tpg220505-bib-0040] Hörtensteiner, S. (2009). Stay‐green regulates chlorophyll and chlorophyll‐binding protein degradation during senescence. Trends in Plant Science, 14(3), 155–162. 10.1016/j.tplants.2009.01.002 19237309

[tpg220505-bib-0041] Hu, Y. , Li, W. C. , Xu, Y. Q. , Li, G. J. , Liao, Y. , & Fu, F.‐L. (2009). Differential expression of candidate genes for lignin biosynthesis under drought stress in maize leaves. Journal of Applied Genetics, 50, 213–223. 10.1007/BF03195675 19638676

[tpg220505-bib-0042] IBPGR and ICRISAT . (1993). Descriptors for sorghum [Sorghum bicolor (L.) Moench] . IBPGR, ICRISAT.

[tpg220505-bib-0043] Jafari, A. , Paknejad, F. , & AL‐Ahmadi , M. J. (2009). Evaluation of selection indices for drought tolerance of corn (*Zea mays* L.) hybrids. International Journal of Plant Production, 3(4), 33–38.

[tpg220505-bib-0044] Jordan, D. R. , Hunt, C. H. , Cruickshank, A. W. , Borrell, A. K. , & Henzell, R. G. (2012). The relationship between the stay‐green trait and grain yield in elite sorghum hybrids grown in a range of environments. Crop Science, 52(3), 1153–1161. 10.2135/cropsci2011.06.0326

[tpg220505-bib-0045] Kam, J. , Gresshoff, P. , Shorter, R. , & Xue, G.‐P. (2007). Expression analysis of RING zinc finger genes from *Triticum aestivum* and identification of TaRZF70 that contains four RING‐H2 domains and differentially responds to water deficit between leaf and root. Plant Science, 173(6), 650–659. 10.1016/j.plantsci.2007.09.001

[tpg220505-bib-0046] Kamal, N. M. , Gorafi, Y. S. A. , Tsujimoto, H. , & Ghanim, A. M. A. (2018). Stay‐green QTLs response in adaptation to post‐flowering drought depends on the drought severity. BioMed Research International, 2018, Article 7082095. 10.1155/2018/7082095 30584537 PMC6280221

[tpg220505-bib-0047] Kassambara, A. , & Mundt, F. (2020). *Factoextra: Extract and visualize the results of multivariate data analyses* (R Package Version 1.0.7). https://CRAN.R-project.org/package=factoextra

[tpg220505-bib-0113] Khalilzade, G. , & Karbalai‐Khiavi, H. (2002). Investigation of drought and heat stress on advanced lines of durum wheat. In Proceedings of the 7th Iranian Congress of Crop Sciences (pp. 563–564). Crop Science Society of Iran.

[tpg220505-bib-0048] Kebede, H. , Subudhi, P. K. , Rosenow, D. T. , & Nguyen, H. T. (2001). Quantitative trait loci influencing drought tolerance in grain sorghum (*Sorghum bicolor* L. Moench). Theoretical and Applied Genetics, 103(2–3), 266–276. 10.1007/s001220100541

[tpg220505-bib-0049] Kilian, A. , Wenzl, P. , Huttner, E. , Carling, J. , Xia, L. , Blois, H. , Caig, V. , Heller‐Uszynska, K. , Jaccoud, D. , Hopper, C. , Aschenbrenner‐Kilian, M. , Evers, M. , Peng, K. , Cayla, C. , Hok, P. , & Uszynski, G. (2012). Diversity arrays technology: A generic genome profiling technology on open platforms. In F. Pompanon , & A. Bonin (Eds.), Data production and analysis in population genomics (Vol. 888, pp. 67–89). Methods in molecular biology. Springer. 10.1007/978-1-61779-870-2_5 22665276

[tpg220505-bib-0050] Kim, J. H. , & Kim, W. T. (2013). The Arabidopsis RING E3 ubiquitin ligase AtAIRP3/LOG2 participates in positive regulation of high‐salt and drought stress responses. Plant Physiology, 162(3), 1733–1749. 10.1104/pp.113.220103 23696092 PMC3707541

[tpg220505-bib-0051] Kim, M.‐H. , Cho, J.‐S. , Park, E.‐J. , Lee, H. , Choi, Y.‐I. , Bae, E.‐K. , Han, K.‐H. , & Ko, J.‐H. (2020). Overexpression of a poplar Ring‐H2 zinc finger, *Ptxerico*, confers enhanced drought tolerance via reduced water loss and ion leakage in *Populus* . International Journal of Molecular Sciences, 21(24), 9454. 10.3390/ijms21249454 33322558 PMC7764267

[tpg220505-bib-0052] Kuznetsova, A. , Brockhoff, P. B. , & Christensen, R. H. B. (2017). lmerTest package: Tests in linear mixed effects models. Journal of Statistical Software, 82(13), 1–26. 10.18637/jss.v082.i13

[tpg220505-bib-0053] Lencina, F. , Landau, A. M. , Petterson, M. E. , Pacheco, M. G. , Kobayashi, K. , & Prina, A. R. (2019). The *rpl23* gene and pseudogene are hotspots of illegitimate recombination in barley chloroplast mutator seedlings. Scientific Reports, 9(1), Article 9960. 10.1038/s41598-019-46321-6 31292475 PMC6620283

[tpg220505-bib-0054] Li, H. , Han, X. , Liu, X. , Zhou, M. , Ren, W. , Zhao, B. , Ju, C. , Liu, Y. , & Zhao, J. (2019). A leucine‐rich repeat‐receptor‐like kinase gene *SbER2–1* from sorghum (*Sorghum bicolor* L.) confers drought tolerance in maize. BMC Genomics, 20(1), Article 737. 10.1186/s12864-019-6143-x 31615416 PMC6794760

[tpg220505-bib-0055] Li, Y. , & Wei, K. (2020). Comparative functional genomics analysis of cytochrome P450 gene superfamily in wheat and maize. BMC Plant Biology, 20, 1–22. 32122306 10.1186/s12870-020-2288-7 PMC7052972

[tpg220505-bib-0056] Liu, H. , Zhang, H. , Yang, Y. , Li, G. , Yang, Y. , Wang, X. E. , Basnayake, B. M. V. S. , Li, D. , & Song, F. (2008). Functional analysis reveals pleiotropic effects of rice RING‐H2 finger protein gene OsBIRF1 on regulation of growth and defense responses against abiotic and biotic stresses. Plant Molecular Biology, 68, 17–30. 10.1007/s11103-008-9349-x 18496756

[tpg220505-bib-0057] Liu, S. , Yang, R. , Liu, M. , Zhang, S. , Yan, K. , Yang, G. , Huang, J. , Zheng, C. , & Wu, C. (2020). PLATZ2 negatively regulates salt tolerance in Arabidopsis seedlings by directly suppressing the expression of the CBL4/SOS3 and CBL10/SCaBP8 genes. Journal of Experimental Botany, 71(18), 5589–5602. 10.1093/jxb/eraa259 32453821

[tpg220505-bib-0058] Liu, Y. , Li, C. , Qin, A. , Deng, W. , Chen, R. , Yu, H. , Wang, Y. , Song, J. , & Zeng, L. (2024). Genome‐wide identification and transcriptome profiling expression analysis of the *U‐box* E3 ubiquitin ligase gene family related to abiotic stress in maize (*Zea mays* L.). BMC Genomics, 25(1), Article 132. 10.1186/s12864-024-10040-8 38302871 PMC10832145

[tpg220505-bib-0059] Mace, E. , Innes, D. , Hunt, C. , Wang, X. , Tao, Y. , Baxter, J. , Hassall, M. , Hathorn, A. , & Jordan, D. (2019). The sorghum QTL atlas: A powerful tool for trait dissection, comparative genomics and crop improvement. Theoretical and Applied Genetics, 132, 751–766. 10.1007/s00122-018-3212-5 30343386

[tpg220505-bib-0060] Maina, F. , Harou, A. , Hamidou, F. , & Morris, G. P. (2022). Genome‐wide association studies identify putative pleiotropic locus mediating drought tolerance in sorghum. Plant Direct, 6(6), e413. 10.1002/pld3.413 35774626 PMC9219007

[tpg220505-bib-0061] Mandaokar, A. , & Browse, J. (2009). MYB108 acts together with MYB24 to regulate jasmonate‐mediated stamen maturation in Arabidopsis. Plant Physiology, 149(2), 851–862. 10.1104/pp.108.132597 19091873 PMC2633834

[tpg220505-bib-0062] Masle, J. , Gilmore, S. R. , & Farquhar, G. D. (2005). The *ERECTA* gene regulates plant transpiration efficiency in *Arabidopsis* . Nature, 436(7052), 866–870. 10.1038/nature03835 16007076

[tpg220505-bib-0063] Menamo, T. , Borrell, A. K. , Mace, E. , Jordan, D. R. , Tao, Y. , Hunt, C. , & Kassahun, B. (2023). Genetic dissection of root architecture in Ethiopian sorghum landraces. Theoretical and Applied Genetics, 136(10), Article 209. 10.1007/s00122-023-04457-0 37715848

[tpg220505-bib-0064] Menezes, C. B. D. , Ticona‐Benavente, C. A. , Tardin, F. D. , Cardoso, M. J. , Bastos, E. A. , Nogueira, D. W. , Portugal, A. F. , Santos, C. V. , & Schaffert, R. E. (2014). Selection indices to identify drought‐tolerant grain sorghum cultivars. Genetics and Molecular Research, 13(4), 9817–9827. 25501191 10.4238/2014.November.27.9

[tpg220505-bib-0065] Mitra, J. (2001). Genetics and genetic improvement of drought resistance in crop plants. Current Science, 80, 758–763.

[tpg220505-bib-0066] Mizutani, M. , & Ohta, D. (2010). Diversification of P450 genes during land plant evolution. Annual Review of Plant Biology, 61, 291–315. 10.1146/annurev-arplant-042809-112305 20192745

[tpg220505-bib-0067] Moin, M. , Bakshi, A. , Madhav, M. S. , & Kirti, P. B. (2017). Expression profiling of ribosomal protein gene family in dehydration stress responses and characterization of transgenic rice plants overexpressing *RPL23A* for water‐use efficiency and tolerance to drought and salt stresses. Frontiers in Chemistry, 5, Article 97. 10.3389/fchem.2017.00097 29184886 PMC5694489

[tpg220505-bib-0068] Mwadzingeni, L. , Shimelis, H. , Dube, E. , Laing, M. D. , & Tsilo, T. J. (2016). Breeding wheat for drought tolerance: Progress and technologies. Journal of Integrative Agriculture, 15(5), 935–943. 10.1016/S2095-3119(15)61102-9

[tpg220505-bib-0069] Oh, J. E. , Kwon, Y. , Kim, J. H. , Noh, H. , Hong, S.‐W. , & Lee, H. (2011). A dual role for MYB60 in stomatal regulation and root growth of *Arabidopsis thaliana* under drought stress. Plant Molecular Biology, 77, 91–103. 10.1007/s11103-011-9796-7 21637967

[tpg220505-bib-0070] Paes de Camargo, M. B. , & Hubbard, K. G. (1999). Drought sensitivity indices for a sorghum crop. Journal of Production Agriculture, 12(2), 312–316. 10.2134/jpa1999.0312

[tpg220505-bib-0071] Pandian, B. A. , Sathishraj, R. , Djanaguiraman, M. , Prasad, P. V. V. , & Jugulam, M. (2020). Role of cytochrome P450 enzymes in plant stress response. Antioxidants, 9(5), 454. 10.3390/antiox9050454 32466087 PMC7278705

[tpg220505-bib-0112] Pariyar, S. R. , Nagel, K. A. , Lentz, J. , Galinski, A. , Wilhelm, J. , Putz, A. , Adels, S. , Heinz, K. , Frohberg, C. , & Watt, M. (2021). Variation in root system architecture among the founder parents of two 8‐way MAGIC wheat populations for selection in breeding. Agronomy, 11(12), 2452. 10.3390/agronomy11122452

[tpg220505-bib-0072] Rakitsch, B. , Lippert, C. , Stegle, O. , & Borgwardt, K. (2013). A Lasso multi‐marker mixed model for association mapping with population structure correction. Bioinformatics, 29(2), 206–214. 10.1093/bioinformatics/bts669 23175758

[tpg220505-bib-0073] Rama Reddy, N. R. , Ragimasalawada, M. , Sabbavarapu, M. M. , Nadoor, S. , & Patil, J. V. (2014). Detection and validation of stay‐green QTL in post‐rainy sorghum involving widely adapted cultivar, M35‐1 and a popular stay‐green genotype B35. BMC Genomics, 15(1), Article 909. 10.1186/1471-2164-15-909 25326366 PMC4219115

[tpg220505-bib-0074] Ren, W.‐L. , Wen, Y.‐J. , Dunwell, J. M. , & Zhang, Y.‐M. (2018). pKWmEB: Integration of Kruskal–Wallis test with empirical Bayes under polygenic background control for multi‐locus genome‐wide association study. Heredity, 120(3), 208–218. 10.1038/s41437-017-0007-4 29234158 PMC5836593

[tpg220505-bib-0075] Rogers, S. O. , & Bendich, A. J. (1985). Extraction of DNA from milligram amounts of fresh, herbarium and mummified plant tissues. Plant Molecular Biology, 5, 69–76. 10.1007/BF00020088 24306565

[tpg220505-bib-0076] Rosielle, A. A. , & Hamblin, J. (1981). Theoretical aspects of selection for yield in stress and non‐stress environment. Crop Science, 21(6), 943–946. 10.2135/cropsci1981.0011183X002100060033x

[tpg220505-bib-0077] Sabadin, P. K. , Malosetti, M. , Boer, M. P. , Tardin, F. D. , Santos, F. G. , Guimarães, C. T. , Gomide, R. L. , Andrade, C. L. T. , Albuquerque, P. E. P. , Caniato, F. F. , Mollinari, M. , Margarido, G. R. A. , Oliveira, B. F. , Schaffert, R. E. , Garcia, A. A. F. , Van Eeuwijk, F. A. , & Magalhaes, J. V. (2012). Studying the genetic basis of drought tolerance in sorghum by managed stress trials and adjustments for phenological and plant height differences. Theoretical and Applied Genetics, 124(8), 1389–1402. 10.1007/s00122-012-1795-9 22297563

[tpg220505-bib-0078] Schippers, J. H. M. , & Mueller‐Roeber, B. (2010). Ribosomal composition and control of leaf development. Plant Science, 179(4), 307–315. 10.1016/j.plantsci.2010.06.012

[tpg220505-bib-0079] Schneider, K. A. , Rosales‐Serna, R. , Ibarra‐Perez, F. , Cazares‐Enriquez, B. , Acosta‐Gallegos, J. A. , Ramirez‐Vallejo, P. , Wassimi, N. , & Kelly, J. D. (1997). Improving common bean performance under drought stress. Crop Science, 37(1), 43–50. 10.2135/cropsci1997.0011183X003700010007x

[tpg220505-bib-0080] Shin, R. , Burch, A. Y. , Huppert, K. A. , Tiwari, S. B. , Murphy, A. S. , Guilfoyle, T. J. , & Schachtman, D. P. (2007). The *Arabidopsis* transcription factor MYB77 modulates auxin signal transduction. The Plant Cell, 19(8), 2440–2453. 10.1105/tpc.107.050963 17675404 PMC2002618

[tpg220505-bib-0081] Shiraku, M. L. , Magwanga, R. O. , Cai, X. , Kirungu, J. N. , Xu, Y. , Mehari, T. G. , Hou, Y. , Wang, Y. , Wang, K. , Peng, R. , Zhou, Z. , & Liu, F. (2021). Knockdown of 60S ribosomal protein L14‐2 reveals their potential regulatory roles to enhance drought and salt tolerance in cotton. Journal of Cotton Research, 4(1), Article 27. 10.1186/s42397-021-00102-7

[tpg220505-bib-0082] Shpak, E. D. (2013). Diverse roles of *ERECTA* family genes in plant development. Journal of Integrative Plant Biology, 55(12), 1238–1250. 10.1111/jipb.12108 24016315

[tpg220505-bib-0083] Shumayla , Sharma, S. , Pandey, A. K. , Singh, K. , & Upadhyay, S. K. (2016). Molecular characterization and global expression analysis of lectin receptor kinases in bread wheat (*Triticum aestivum*). PLoS ONE, 11(4), e0153925. 10.1371/journal.pone.0153925 27111449 PMC4844157

[tpg220505-bib-0084] Srinivas, G. , Satish, K. , Madhusudhana, R. , Nagaraja Reddy, R. , Murali Mohan, S. , & Seetharama, N. (2009). Identification of quantitative trait loci for agronomically important traits and their association with genic‐microsatellite markers in sorghum. Theoretical and Applied Genetics, 118(8), 1439–1454. 10.1007/s00122-009-0993-6 19274449

[tpg220505-bib-0085] Stichler, C. , & Fipps, G. (2003). Irrigating sorghum in South‐ and South‐Central Texas. Texas A&M University.

[tpg220505-bib-0086] Subudhi, P. K. , Rosenow, D. T. , & Nguyen, H. T. (2000). Quantitative trait loci for the stay green trait in sorghum (*Sorghum bicolor* L. Moench): Consistency across genetic backgrounds and environments. Theoretical and Applied Genetics, 101(5‐6), 733–741. 10.1007/s001220051538

[tpg220505-bib-0087] Sukumaran, S. , Li, X. , Li, X. , Zhu, C. , Bai, G. , Perumal, R. , Tuinstra, M. R. , Prasad, P. V. V. , Mitchell, S. E. , Tesso, T. T. , & Yu, J. (2016). QTL mapping for grain yield, flowering time, and stay‐green traits in sorghum with genotyping‐by‐sequencing markers. Crop Science, 56(4), 1429–1442. 10.2135/cropsci2015.02.0097

[tpg220505-bib-0088] Tamba, C. L. , Ni, Y.‐L. , & Zhang, Y.‐M. (2017). Iterative sure independence screening EM‐Bayesian LASSO algorithm for multi‐locus genome‐wide association studies. PLoS Computational Biology, 13(1), e1005357. 10.1371/journal.pcbi.1005357 28141824 PMC5308866

[tpg220505-bib-0089] Tamba, C. L. , & Zhang, Y.‐M. (2018). A fast mrMLM algorithm for multi‐locus genome‐wide association studies. BioRxiv. 10.1101/341784

[tpg220505-bib-0090] Tamiru, M. , Undan, J. R. , Takagi, H. , Abe, A. , Yoshida, K. , Undan, J. Q. , Natsume, S. , Uemura, A. , Saitoh, H. , Matsumura, H. , Urasaki, N. , Yokota, T. , & Terauchi, R. (2015). A cytochrome P450, OsDSS1, is involved in growth and drought stress responses in rice (*Oryza sativa* L.). Plant Molecular Biology, 88, 85–99. 10.1007/s11103-015-0310-5 25800365

[tpg220505-bib-0091] Thomas, H. , & Ougham, H. (2014). The stay‐green trait. Journal of Experimental Botany, 65(14), 3889–3900. 10.1093/jxb/eru037 24600017

[tpg220505-bib-0092] Tyagi, S. , & Upadhyay, S. K. (2023). Role of leucine‐rich repeat receptor‐like kinases in abiotic and biotic stress responses in plants. In S. K. Upadhyay & Shumayla (Eds.), Plant receptor‐like kinases (pp. 239–255). Academic Press.

[tpg220505-bib-0093] Uluisik, S. , Kıyak, A. , Kurt, F. , & Filiz, E. (2022). *STAY‐GREEN* (*SGR*) genes in tomato (*Solanum lycopersicum*): Genome‐wide identification, and expression analyses reveal their involvements in ripening and salinity stress responses. Horticulture, Environment, and Biotechnology, 63(4), 557–569. 10.1007/s13580-022-00419-5

[tpg220505-bib-0115] Wadan, A. , Abd El Shafi, M. , Gheith, E. , & Suleiman, H. (2015). Using different statistical procedures for evaluating drought tolerance indices of bread wheat genotypes. Advance in Agriculture and Biology, 4(41), 19–30.

[tpg220505-bib-0094] Wang, H. , Chen, G. , Zhang, H. , Liu, B. , Yang, Y. , Qin, L. , Chen, E. , & Guan, Y. (2014). Identification of QTLs for salt tolerance at germination and seedling stage of *Sorghum bicolor* L. Moench. Euphytica, 196(1), 117–127. 10.1007/s10681-013-1019-7

[tpg220505-bib-0095] Wang, S.‐B. , Feng, J.‐Y. , Ren, W.‐L. , Huang, B. , Zhou, L. , Wen, Y.‐J. , Zhang, J. , Dunwell, J. M. , Xu, S. , & Zhang, Y.‐M. (2016). Improving power and accuracy of genome‐wide association studies via a multi‐locus mixed linear model methodology. Scientific Reports, 6(1), Article 19444. 10.1038/srep19444 26787347 PMC4726296

[tpg220505-bib-0096] Wang, X. , Mace, E. , Tao, Y. , Cruickshank, A. , Hunt, C. , Hammer, G. , & Jordan, D. (2020). Large‐scale genome‐wide association study reveals that drought‐induced lodging in grain sorghum is associated with plant height and traits linked to carbon remobilisation. Theoretical and Applied Genetics, 133, 3201–3215. 10.1007/s00122-020-03665-2 32833037

[tpg220505-bib-0097] Wang, X. , Niu, Y. , & Zheng, Y. (2021). Multiple functions of MYB transcription factors in abiotic stress responses. International Journal of Molecular Sciences, 22(11), 6125. 10.3390/ijms22116125 34200125 PMC8201141

[tpg220505-bib-0098] Wen, Y.‐J. , Zhang, H. , Ni, Y.‐L. , Huang, B. , Zhang, J. , Feng, J.‐Y. , Wang, S.‐B. , Dunwell, J. M. , Zhang, Y.‐M. , & Wu, R. (2018). Methodological implementation of mixed linear models in multi‐locus genome‐wide association studies. Briefings in Bioinformatics, 19(4), 700–712. 10.1093/bib/bbw145 28158525 PMC6054291

[tpg220505-bib-0099] Wondimu, Z. , Dong, H. , Paterson, A. H. , Worku, W. , & Bantte, K. (2023). Genome‐wide association study reveals genomic loci influencing agronomic traits in Ethiopian sorghum (*Sorghum bicolor* (L.) Moench) landraces. Molecular Breeding, 43(5), Article 32. 10.1007/s11032-023-01381-5 37312746 PMC10248676

[tpg220505-bib-0100] Wu, F. , Sheng, P. , Tan, J. , Chen, X. , Lu, G. , Ma, W. , Heng, Y. , Lin, Q. , Zhu, S. , Wang, J. , Wang, J. , Guo, X. , Zhang, X. , Lei, C. , & Wan, J. (2015). Plasma membrane receptor‐like kinase leaf panicle 2 acts downstream of the DROUGHT AND SALT TOLERANCE transcription factor to regulate drought sensitivity in rice. Journal of Experimental Botany, 66(1), 271–281. 10.1093/jxb/eru417 25385766 PMC4265162

[tpg220505-bib-0101] Xin, Y. , Gao, L. , Hu, W. , Gao, Q. , Yang, B. , Zhou, J. , & Xu, C. (2022). Genome‐wide association study based on plant height and drought‐tolerance indices reveals two candidate drought‐tolerance genes in sweet sorghum. Sustainability, 14(21), 14339. 10.3390/su142114339

[tpg220505-bib-0102] Xing, H. T. , Guo, P. , Xia, X. L. , & Yin, W. L. (2011). PdERECTA, a leucine‐rich repeat receptor‐like kinase of poplar, confers enhanced water use efficiency in *Arabidopsis* . Planta, 234, 229–241. 10.1007/s00425-011-1389-9 21399949

[tpg220505-bib-0103] Xu, W. , Subudhi, P. K. , Crasta, O. R. , Rosenow, D. T. , Mullet, J. E. , & Nguyen, H. T. (2000). Molecular mapping of QTLs conferring stay‐green in grain sorghum (*Sorghum bicolor* L. Moench). Genome, 43(3), 461–469. 10.1139/g00-003 10902709

[tpg220505-bib-0104] Zeng, D.‐E. , Hou, P. , Xiao, F. , & Liu, Y. (2014). Overexpressing a novel RING‐H2 finger protein gene, OsRHP1, enhances drought and salt tolerance in rice (*Oryza sativa* L.). Journal of Plant Biology, 57, 357–365. 10.1007/s12374-013-0481-z

[tpg220505-bib-0105] Zhang, J. , Feng, J.‐Y. , Ni, Y.‐L. , Wen, Y.‐J. , Niu, Y. , Tamba, C. L. , Yue, C. , Song, Q. , & Zhang, Y.‐M. (2017). pLARmEB: Integration of least angle regression with empirical Bayes for multilocus genome‐wide association studies. Heredity, 118(6), 517–524. 10.1038/hdy.2017.8 28295030 PMC5436030

[tpg220505-bib-0106] Zhang, Y. , Fan, X. , Liang, D. , Guo, Q. , Zhang, X. , Nie, M. , Li, C. , Meng, S. , Zhang, X. , Xu, P. , Guo, W. , Wang, H. , Liu, Q. , & Wu, Y. (2023). The identification of a yield‐related gene controlling multiple traits using GWAS in sorghum (*Sorghum bicolor* L.). Plants, 12(7), 1557. 10.3390/plants12071557 37050183 PMC10097259

[tpg220505-bib-0107] Zhang, Y.‐W. , Tamba, C. L. , Wen, Y.‐J. , Li, P. , Ren, W.‐L. , Ni, Y.‐L. , Gao, J. , & Zhang, Y.‐M. (2020). mrMLM v4. 0.2: An R platform for multi‐locus genome‐wide association studies. Genomics, Proteomics & Bioinformatics, 18(4), 481–487. 10.1016/j.gpb.2020.06.006 PMC8242264 33346083

[tpg220505-bib-0108] Zhao, J. , Zheng, L. , Wei, J. , Wang, Y. , Chen, J. , Zhou, Y. , Chen, M. , Wang, F. , Ma, Y. , & Xu, Z.‐S. (2022). The soybean PLATZ transcription factor GmPLATZ17 suppresses drought tolerance by interfering with stress‐associated gene regulation of GmDREB5. The Crop Journal, 10(4), 1014–1025. 10.1016/j.cj.2022.03.009

[tpg220505-bib-0109] Zheng, R. , Deng, M. , Lv, D. , Tong, B. , Liu, Y. , & Luo, H. (2023). Combined BSA‐seq and RNA‐seq reveal genes associated with the visual stay‐green of maize (*Zea mays* L.). International Journal of Molecular Sciences, 24(24), 17617. 10.3390/ijms242417617 38139444 PMC10744276

[tpg220505-bib-0110] Zhong, H. , Liu, S. , Sun, T. , Kong, W. , Deng, X. , Peng, Z. , & Li, Y. (2021). Multi‐locus genome‐wide association studies for five yield‐related traits in rice. BMC Plant Biology, 21(1), Article 364. 10.1186/s12870-021-03146-8 34376143 PMC8353822

