# Supplementary material for: Multi‐locus genome‐wide association study for grain yield and drought tolerance indices in sorghum accessions
Source: Plant Genome. 2024 Sep 10;17(4):e20505. doi: 10.1002/tpg2.20505 (PMC11628898; doi:10.1002/tpg2.20505)
Supplement: Supplementary file 3 [file TPG2-17-e20505-s005.docx]

Supplementary Figure S1: dendrogram of 216 sorghum accessions based on drought induces at Melkassa & Werer experimental site.
