# Supplementary material for: Multi‐locus genome‐wide association study for grain yield and drought tolerance indices in sorghum accessions
Source: Plant Genome. 2024 Sep 10;17(4):e20505. doi: 10.1002/tpg2.20505 (PMC11628898; doi:10.1002/tpg2.20505)
Supplement: Supplementary file 6 [file TPG2-17-e20505-s008.docx]

Supplementary Table S3: List of accessions per cluster in Melkassa

| Cluster | Proportion | List of accessions in the clusters |
| --- | --- | --- |
| C-I | 0.31 | Acc#9577; Acc#220259; Acc#220267; Acc#220269; Acc#220272; Acc#220275; Acc#220277; Acc#220278; Acc#234066; Acc#234101; Acc#235793; Acc#235794; Acc#235807; Acc#23644; Acc#235808; Acc#235817; Acc#1127; Acc#2262; Acc#23650; Acc#2814; Acc#3443; Acc#3675; Acc#5622; Acc#6094; Acc#7463; Acc#9830; Acc#10234; Acc#10978; Acc#11119; Acc#15428; Acc#28545; Acc#20205; Acc#20387; Acc#20713; Acc#28547; Acc#20762; Acc#23178; Acc#28550; Acc#25596; Acc#28551; Acc#27599; Acc#29375; Acc#28556; Acc#29977; Acc#30001; Acc#30318; Acc#31681; Acc#69212; Acc#30469; Malt sorghum#384; Malt sorghum#385; Malt sorghum#391; Malt sorghum#392; Kem Kem; Acc#69568; Acc#69571; Acc#69573; Acc#220010; Acc#220012; Acc#220237; Acc#220241; Acc#220242; Acc#220243; Acc#220244; Acc#220249; Acc#220250; & Acc#220256 |
| C-II | 0.38 | Acc#220260; Acc#220261; Acc#220262; Acc#220264; Acc#220265; Acc#220266; Acc#220270; Acc#220273; Acc#220274; Acc#220276; Acc#220279; Acc#220281; Acc#234070; Acc#234102; Acc#234113; Acc#235790; Acc#235812; Acc#238431; Acc#238442; Acc#238444; Acc#238447; Acc#9600; Acc#2848; Acc#3121; Acc#6193; Acc#6723; Acc#6928; Acc#7125; Acc#8218; Acc#9713; Acc#10876; Acc#15443; Acc#15752; Acc#19126; Acc#19262; Acc#20351; Acc#20665; Acc#20681; Acc#20700; Acc#20710; Acc#20727; Acc#20842; Acc#15964; Acc#28548; Acc#22040; Acc#22074; Acc#22291; Acc#22506; Acc#24083; Acc#25442; Acc#25702; Acc#26110; Acc#27287; Acc#28740; Acc#29310; Acc#29409; Acc#29876; Acc#30317; Acc#30175; Acc#28557; Acc#30503; Acc#30619; Acc#31693; Acc#31852; Acc#32087; Acc#36633; Malt sorghum#389; Acc#28688; Acc#211022; Acc#220227; Acc#220236; Acc#220238; Acc#220240; Acc#220246; Acc#220247; Acc#220248; Acc#220251; Acc#220253; Acc#220254; Acc#220255; & Acc#220257 |
| C-III | 0.24 | Acc#227085; Acc#227091; Acc#230065; Acc#231230; Acc#234089; Acc#234110; Acc#235789; Acc#235791; Acc#235792; Acc#235798; Acc#235803; Acc#235804; Acc#235810; Acc#235811; Acc#235813; Acc#235814; Acc#238440; Acc#2398; Acc#2416; Acc#2787; Acc#3073; Acc#9911; Acc#13845; Acc#14963; Acc#15526; Acc#16044; Acc#27919; Acc#19053; Acc#19847; Acc#20697; Acc#20749; Acc#22239; Acc#22330; Acc#22334; Acc#28549; Acc#23053; Acc#23601; Acc#26833; Acc#28991; Acc#29911; Acc#30898; Acc#33173; Malt sorghum#386 ; Malt sorghum#387; Malt sorghum#390; Wedi Aker; B-35; Acc#220001; Acc#220013; Acc#220018; Acc#19627; & Acc#220252 |
| C-IV | 0.07 | Acc#23635; Acc#220268; Acc#23637; Acc#222285; Acc#222888, Acc#234115; Acc#239130; Acc#3583; **Acc#28546**; Acc#36524; Melkam; Acc#216736; **Acc#216739**; Acc#216744; & Acc#19615 |

Supplementary Table S3: List of accessions per cluster in Werer

| Cluster | Pro. | List of accessions in the clusters |
| --- | --- | --- |
| C-I | 0.5 | Acc#9577; Acc#220259; Acc#220260; Acc#220261; Acc#220262; Acc#220269; Acc#220270; Acc#220272; Acc#220273; Acc#220275; Acc#220276; Acc#220277; Acc#220278; Acc#23637; Acc#220281; Acc#222285; Acc#227091; Acc#230065; Acc#234113; Acc#234115; Acc#235790; Acc#235792; Acc#235793; Acc#235794; Acc#235798; Acc#235808; Acc#235810; Acc#235814; Acc#238440; Acc#238444; Acc#1127; Acc#23650; Acc#2398; Acc#2416; Acc#2787; Acc#2814; Acc#2848; Acc#3073; Acc#3443; Acc#3583; Acc#5622; Acc#6094; Acc#6193; Acc#6928; Acc#9830; Acc#9911; Acc#10234; Acc#10876; Acc#10978; Acc#11119; Acc#15526; Acc#16044; Acc#20351; Acc#20387; Acc#20681; Acc#20697; Acc#20700; Acc#20713; Acc#28547; Acc#20749; Acc#20762; Acc#28548; Acc#22040; Acc#22291; Acc#22334; Acc#28549; Acc#24083; Acc#25596; Acc#27599; Acc#29409; Acc#28556; Acc#28557; Acc#30898; Acc#31852; Acc#32087; Acc#33173; Acc#69212; Acc#36633; Acc#30469; Acc#36524; Malt sorghum#384; Malt sorghum#385; Malt sorghum#387; Malt sorghum#390; Malt sorghum#391; Malt sorghum#392; Acc#69568; Acc#69571; Acc#211022; Acc#220001; Acc#220010; Acc#220012; Acc#19615; Acc#220018; Acc#220236; Acc#220238; Acc#220243; Acc#220244; Acc#220246; Acc#220247; Acc#19627; Acc#220248; Acc#220249; Acc#220250; Acc#220251; Acc#220252; Acc#220254; Acc#220255; & Acc#220256 |
| C-II | 0.30 | Acc#23635; Acc#220264; Acc#220266; Acc#220267; Acc#220268; Acc#220274; Acc#220279; Acc#227085; Acc#234066; Acc#234070; Acc#234101; Acc#234102; Acc#238431; Acc#238442; Acc#238447; Acc#9600; Acc#3121; Acc#3675; Acc#6723; Acc#8218; Acc#9713; Acc#13845; Acc#14963; Acc#15428; Acc#15752; Acc#19053; Acc#28545; Acc#19126; Acc#19262; Acc#20665; Acc#20710; Acc#20727; Acc#20842; Acc#22074; Acc#22330; Acc#22506; Acc#23178; Acc#25442; Acc#25702; Acc#26110; Acc#28551; Acc#27287; Acc#29375; Acc#29876; Acc#30001; Acc#30317; Acc#30318; Acc#30503; Acc#30619; Acc#31681; Acc#31693; Malt sorghum#386; Malt sorghum#389; Wedi Aker; Kem Kem; Acc#28688; Acc#69573; Acc#220013; Acc#220227; Acc#220240; Acc#220241; Acc#220242; Acc#220253; & Acc#220257 |
| C-III | 0.14 | Acc#220265; Acc#231230; Acc#234089; Acc#235791; Acc#235803; Acc#235811; Acc#235812; Acc#235817; Acc#239130; Acc#2262; Acc#7125; Acc#7463; Acc#15443; Acc#27919; Acc#19847; Acc#20205; Acc#15964; Acc#22239; Acc#23053; Acc#23601; Acc#28550; Acc#26833; Acc#28740; Acc#29911; Acc#29977; Acc#30175; Melkam; Acc#216736; Acc#216744; & Acc#220237 |
| C-IV | 0.06 | Acc#222888; Acc#234110; Acc#235789; Acc#235804; Acc#235807; Acc#23644; Acc#235813; **Acc#28546**; Acc#28991; Acc#29310; B-35; & **Acc#216739** |
