# Supplementary material for: Multi‐locus genome‐wide association study for grain yield and drought tolerance indices in sorghum accessions
Source: Plant Genome. 2024 Sep 10;17(4):e20505. doi: 10.1002/tpg2.20505 (PMC11628898; doi:10.1002/tpg2.20505)
Supplement: Supplementary file 7 [file TPG2-17-e20505-s009.docx]

**Supplementary Table S6**: List of significantly associated QTNs at either two locations or at least three drought-induced and co-located regions with previously studied QTLs

| **QTNs** | **QTL Id** | **Population** | **Trait Description** | **LG** | **Start** | **End (v3.0)** | **Publication** |
| --- | --- | --- | --- | --- | --- | --- | --- |
| S1_67598132 | QSTGR1.2 | Advanced Yield Test germplasm set | Stay-green | 1 | 67,191,764 | 67,694,876 | Wang et al. (2014) |
| S10_11382487 | QPNUM10.2 | Diversity research set (n=107) | Panicle number | 10 | 10,520,111 | 13,359,690 | Sakhi et al. (2013) |
| S10_11382487 | QLFAR10.12 | IS2449/IS1488 | Leaf area | 10 | 10,532,053 | 50,312,453 | Phuong et al. (2013) |
| S10_11382487 | QDTFL10.35 | IS2449/IS1488 | Days to flowering | 10 | 10,648,860 | 49,470,823 | Phuong et al. (2013) |
| S5_54698125 & S5_54865070 | QCHLC5.1 | B35/Tx430 | Chlorophyll content | 5 | 10,858,103 | 56,278,757 | Crasta et al. (1999) |
| S5_54698125 & S5_54865070 | QSTGR5.1 | B35/Tx7000 | Stay-green | 5 | 10,879,956 | 58,219,061 | Xu et al. (2000) |
| S5_54698125 & S5_54865070 | QSTGR5.2 | B35/Tx7000 | Stay-green | 5 | 10,879,956 | 58,219,061 | Xu et al. (2000) |
| S5_54698125 & S5_54865070 | QSTGR5.3 | B35/Tx7000 | Stay-green | 5 | 10,923,140 | 62,813,315 | Subudhi et al. (2000) |
| S5_54698125 & S5_54865070 | QSTGR5.4 | B35/Tx7000 | Stay-green | 5 | 11,066,441 | 61,189,149 | Xu et al. (2000) |
| S5_54698125 & S5_54865070 | QCHLC5.2 | M35-1/B35 | Chlorophyll content | 5 | 11,498,149 | 55,431,220 | Reddy et al. (2014) |
| S5_54698125 & S5_54865070 | QDTFL5.16 | IS2449/IS1488 | Days to flowering | 5 | 11,927,145 | 60,526,482 | Phuong et al. (2013) |
| S5_54698125 & S5_54865070 | QSTGR5.5 | SC56/Tx7000 | Stay-green | 5 | 12,788,129 | 61,572,575 | Kebede et al. (2001) |
| S5_54698125 & S5_54865070 | QCHLF5.12 | Diversity set (biomass) (n=194) | Chlorophyll fluorescence | 5 | 13,325,153 | 51,801,797 | Fiedler et al. (2014) |
| S5_54698125 & S5_54865070 | QCHLC5.12 | Tx436/00MN7645 | Chlorophyll content | 5 | 52,630,224 | 64,143,915 | Sukumaran et al. (2016) |
| S6_48187126 | QGLFA6.2 | SC283/BR007 | Green leaf area | 6 | 2,490,610 | 52,124,181 | Sabadin et al. (2012) |
| S6_48187126 | QGLFA6.1 | 296B/IS18551 | Green leaf area | 6 | 46,558,064 | 50,652,065 | Srinivas et al. (2009) |
| S6_48187126 | QTNGL6.1 | M35-1/B35 | Total number of green leaves | 6 | 47,995,582 | 51,437,240 | Reddy et al. (2014) |
