# Supplementary material for: Multi‐locus genome‐wide association study for grain yield and drought tolerance indices in sorghum accessions
Source: Plant Genome. 2024 Sep 10;17(4):e20505. doi: 10.1002/tpg2.20505 (PMC11628898; doi:10.1002/tpg2.20505)
Supplement: Supplementary file 8 [file TPG2-17-e20505-s010.docx]

Supplementary Figure S3: Transition and transversion based on bi-allelic SNP markers. Tv: Transversions; Ts: Transitions; A: Adenine; T: Thymine; G: Guanine; C: Cytosine. The 1st combination nucleotide refers the mutation in individual accession and the 2nd nucleotide combination refers the reference genome nucleotide.
