# Supplementary figures and images for: Multi‐locus genome‐wide association study for grain yield and drought tolerance indices in sorghum accessions
Source: Plant Genome. 2024 Sep 10;17(4):e20505. doi: 10.1002/tpg2.20505 (PMC11628898; doi:10.1002/tpg2.20505)

**
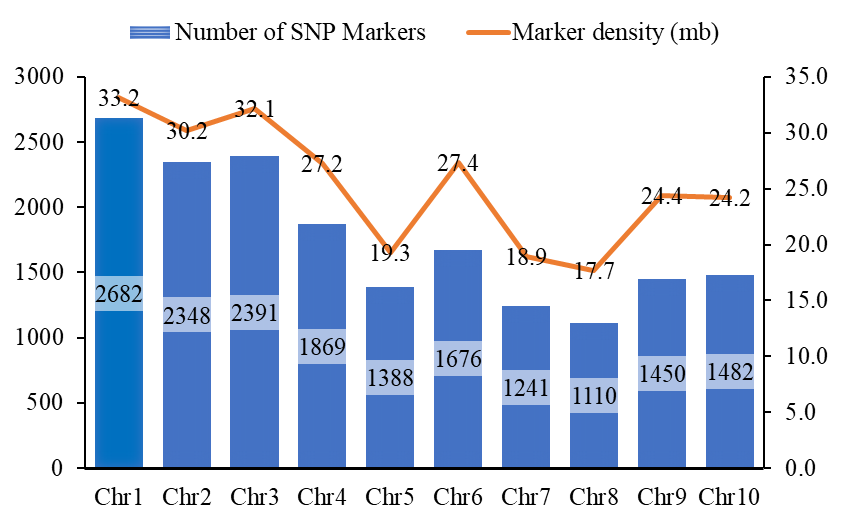

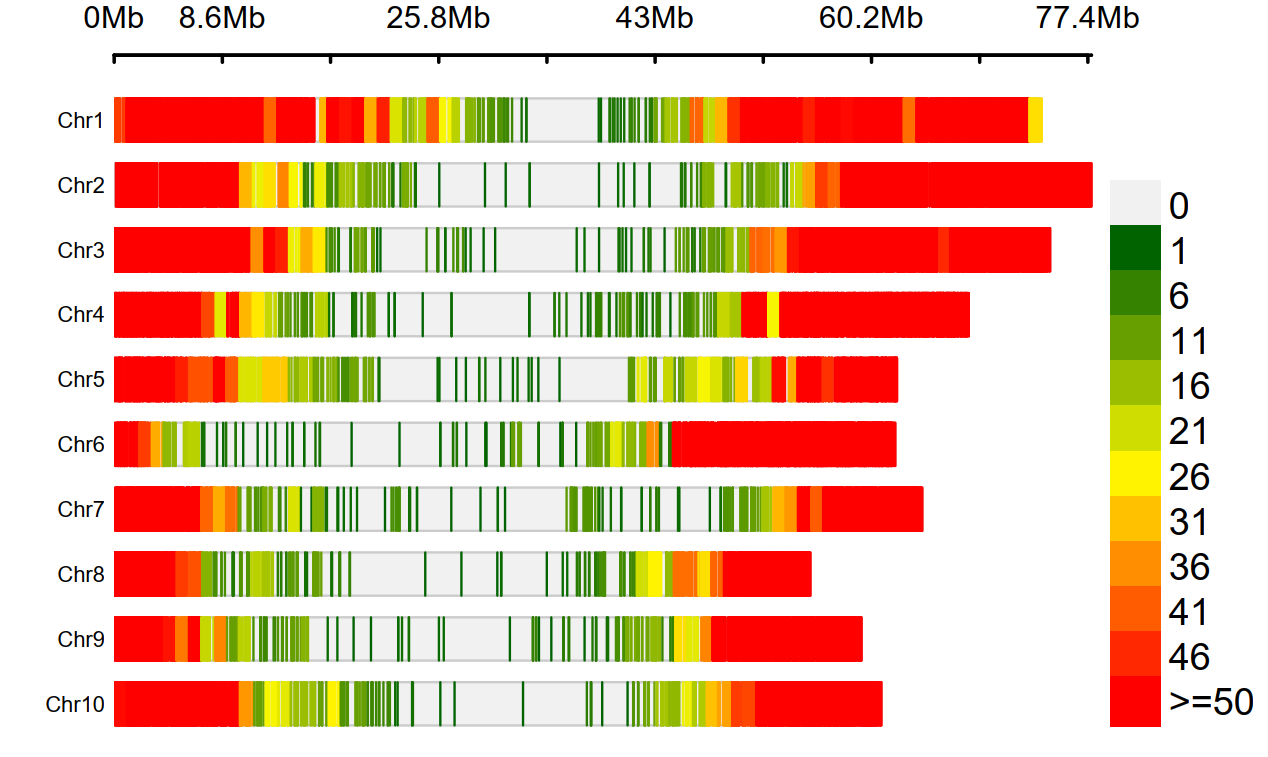
**

Supplementary Figure S2: SNP makers and marker density (Mb) across 10 sorghum chromosomes.

Supplement: Supplementary file 2 — Supplementary Figure S2: SNP makers and marker density (Mb) across 10 sorghum chromosomes. [file TPG2-17-e20505-s004.docx]
