# Supplementary material for: Multi‐locus genome‐wide association study for grain yield and drought tolerance indices in sorghum accessions
Source: Plant Genome. 2024 Sep 10;17(4):e20505. doi: 10.1002/tpg2.20505 (PMC11628898; doi:10.1002/tpg2.20505)
Supplement: Supplementary file 7 — Supplementary Table S4: List of significant QTNs codetected simultaneously by using three or more multilocus GWAS methods for drought indices and using grain yield traits in Melkassa. [file TPG2-17-e20505-s006.docx]

**Supplementary Table S4**: List of significant QTNs codetected simultaneously by using three or more multilocus GWAS methods for drought indices and using grain yield traits in Melkassa

| **Trait** | **QTN** | **Chr** | **Position (bp)** | **LOD score** | **-log10(P)** | **r^2^ (%)** | **Method** |
| --- | --- | --- | --- | --- | --- | --- | --- |
| **Yi** | S1_67598132 | Sb-01 | 67598132 | 3.0-4.8 | 3.7-4.8 | 3.1-7.8 | 1, 2, & 5 |
|  | S1_69125277 | Sb-01 | 69125277 | 3.5-5.1 | 4.2-5.9 | 2.7-11.4 | 1, 2, & 4 |
|  | S7_23456610 | Sb-07 | 23456610 | 3.2-4.5 | 4.0-5.3 | 2.5-7.4 | 2, 4, & 6 |
|  | S10_11382487 | Sb-10 | 11382487 | 5.4-8.0 | 6.3-8.9 | 9.3-21.5 | 3, 4, 5, & 6 |
| **Ys** | S2_1027841 | Sb-02 | 1027841 | 3.9-7.1 | 5.3-8.0 | 5.9-11.8 | 1, 2, 3, 4,5, & 6 |
|  | S5_54698125 | Sb-05 | 54698125 | 3.1-5.2 | 3.9-8.9 | 2.4-10.1 | 1, 2, 3, 4, 5 & 6 |
|  | S5_54865070 | Sb-05 | 54865070 | 3.7-7.8 | 5.2-6.3 | 4.1-8.9 | 3, 4 & 6 |
| **STI** | S1_67598132 | Sb-01 | 67598132 | 3.6-5.6 | 4.5-6.8 | 2.0-8.5 | 2, 4 & 5 |
|  | S5_447153 | Sb-05 | 447153 | 3.1-6.2 | 3.8-7.1 | 1.1-5.1 | 2, 4, & 5 |
|  | S5_54865070 | Sb-05 | 54865070 | 3.1-7.1 | 4.8-5.6 | 0.6-7.0 | 3, 4, & 6 |
|  | S6_1578778 | Sb-06 | 1578778 | 3.2-7.2 | 4.8-5.8 | 0.28-10.7 | 1, 2, 4, & 5 |
|  | S7_62422830 | Sb-07 | 62422830 | 3.0-4.2 | 3.7-5.0 | 0.8-5.6 | 2, 4 & 5 |
| **MP** | S1_67598132 | Sb-01 | 67598132 | 3.3-4.8 | 4.1-5.6 | 1.9-9.5 | 2, 4, & 5 |
|  | S1_70364435 | Sb-01 | 70364435 | 3.2-4.8 | 3.9-5.6 | 2.2-4.9 | 4, 5, & 6 |
|  | S2_9639318 | Sb-02 | 9639318 | 3.3-4.8 | 4.0-5.6 | 1.9-6.6 | 4, 5, & 6 |
|  | S3_60773129 | Sb-03 | 60773129 | 5.8-7.1 | 6.7-8.0 | 3.6-8.2 | 1, 4, & 6 |
|  | S5_54865070 | Sb-05 | 54865070 | 5.7-7.6 | 6.6-8.5 | 4.3-9.8 | 2, 3, 4, 5, & 6 |
|  | S7_23456610 | Sb-07 | 23456610 | 4.9-6.9 | 5.8-7.8 | 7.4-14.5 | 2, 4, & 6 |
| **GMP** | S4_67338619 | Sb-04 | 67338619 | 4.0-5.4 | 4.8-6.2 | 2.6-4.9 | 1, 2, 3, & 4 |
|  | S5_54698125 | Sb-05 | 54698125 | 3.8-5.7 | 4.5-6.5 | 2.2-8.6 | 1, 2, 4, 5, & 6 |
|  | S5_54865070 | Sb-05 | 54865070 | 4.1-7.5 | 4.8-8.4 | 3.1-8.8 | 3, 4, & 6 |
|  | S7_63079783 | Sb-07 | 63079783 | 3.4-5.3 | 4.1-6.1 | 1.9-4.6 | 2, 4, & 5 |
|  | S9_1270591 | Sb-09 | 1270591 | 3.0-3.5 | 3.7-4.3 | 2.2-4.7 | 1, 2, & 5 |
| **HM** | S2_63531635 | Sb-02 | 63531635 | 3.1-5.3 | 3.8-6.1 | 3.9-5.4 | 2, 5, & 6 |
|  | S4_67338619 | Sb-04 | 67338619 | 3.6-7.7 | 4.4-8.6 | 3.0-6.9 | 1, 2, 4, & 6 |
|  | S5_54698125 | Sb-05 | 54698125 | 3.7-6.3 | 4.3-7.1 | 2.6-5.8 | 1,2,3,4, & 5 |
|  | S5_54865070 | Sb-05 | 54865070 | 5.2-10.6 | 6.0-11.6 | 6.2-11.0 | 2, 3, 5, & 6 |
| **YSI** | S10_5995391 | Sb-10 | 5995391 | 4.1-5.5 | 3.0-8.5 | 3.0-8.5 | 1, 2, & 6 |
|  | S3_72667269 | Sb-03 | 72667269 | 3.9-44.5 | 1.4-6.3 | 1.4-6.3 | 1, 2, & 5 |
|  | S7_53804119 | Sb-07 | 53804119 | 4.4-7.1 | 3.7-9.0 | 3.7-8.9 | 1, 2, & 4 |
| **YL** | S2_1027841 | Sb-02 | 1027841 | 4.2-6.1 | 4.9-7.0 | 5.9-13.9 | 1, 3, 4, 5, & 6 |
|  | S2_71825357 | Sb-02 | 71825357 | 3.0-3.3 | 3.7-4.0 | 2.2-6.1 | 1, 3, & 6 |
|  | S5_54698125 | Sb-05 | 54698125 | 3.3-5.2 | 4.0-6.0 | 2.1-10.1 | 1, 4, & 5 |
|  | S5_54865070 | Sb-05 | 54865070 | 4.2-7.1 | 5.0-7.9 | 4.1-8.9 | 2,4, & 6 |

Methods 1–6 include 1=mrMLM, 2=FASTmrMLM, 3=FASTmrEMMA, 4=pLARmEB, 5=pKWmEB, and 6=ISIS EM-BLASSO. r ^2^ (%) the proportion of total phenotypic variance explained by each QTN.
