# Supplementary material for: Multi‐locus genome‐wide association study for grain yield and drought tolerance indices in sorghum accessions
Source: Plant Genome. 2024 Sep 10;17(4):e20505. doi: 10.1002/tpg2.20505 (PMC11628898; doi:10.1002/tpg2.20505)
Supplement: Supplementary file 8 — Supplementary Table 5: List of significant QTNs co‐detected simultaneously by using three or more multi‐locus GWAS methods for drought indices and using grain yield traits in Werer. [file TPG2-17-e20505-s008.docx]

**Supplementary Table 5**: List of significant QTNs co-detected simultaneously by using three or more multi-locus GWAS methods for drought indices and using grain yield traits in Werer

| Trait | QTN | Chr | Position (bp) | LOD score | -log10(P) | r^2^ (%) | Method |
| --- | --- | --- | --- | --- | --- | --- | --- |
| **Yi** | S1_1535802 | Sb-01 | 1535802 | 4.5-6.1 | 5.3-7.0 | 7.1-9.7 | 3, 4, & 5 |
|  | S10_11382487 | Sb-10 | 11382487 | 4.6-6.7 | 5.3-7.6 | 7.9- 28.0 | 3, 5, & 6 |
|  | S3_72141695 | Sb-03 | 72141695 | 4.6-8.3 | 5.4-9.2 | 5.8-11.11 | 1, 2, 3, 4, & 5 |
|  | S4_60778923 | Sb-04 | 60778923 | 3.1-5.4 | 3.8- 6.2 | 3.6-5.8 | 1, 3, & 4 |
| **Ys** | S4_51753172 | Sb-04 | 51753172 | 3.2-5.1 | 3.9-5.9 | 1.1-6.8 | 2, 4, 5, & 6 |
|  | S6_48187126 | Sb-06 | 48187126 | 3.0-5.3 | 3.7-6.2 | 0.8-12.70 | 4, 5, & 6 |
|  | S7_58489245 | Sb-07 | 58489245 | 3.3-5.1 | 4.2- 5.9 | 1.0-7.2 | 2, 4, 5, & 6 |
| **STI** | S6_48187126 | Sb-06 | 48187126 | 6.4-9.9 | 8.0-10.9 | 1.8-14.8 | 1, 4, 5, & 6 |
| **MP** | S1_1535802 | Sb-01 | 1535802 | 3.7-10.3 | 4.5- 11.3 | 1.8-14.7 | 1, 2, 3, 4, 5, & 6 |
|  | S10_11382487 | Sb-10 | 11382487 | 4.8-9.2 | 5.6- 10.2 | 8.8-32.9 | 1, 3, 4, & 6 |
|  | S2_65999614 | Sb-02 | 65999614 | 3.7-6.2 | 4.5- 7.1 | 3.2-9.1 | 1, 2, 3, & 5 |
|  | S6_48187126 | Sb-06 | 48187126 | 3.1-5.1 | 4.0- 5.9 | 1.5-7.3 | 1, 4, & 6 |
|  | S6_54325132 | Sb-06 | 54325132 | 3.1-4.0 | 4.0- 4.7 | 2.4 – 6.4 | 2, 3, & 5 |
| **GMP** | S1_1535802 | Sb-01 | 1535802 | 4.6-7.6 | 5.4- 8.5 | 3.4-9.2 | 1, 2, 3, & 4 |
|  | S2_65999614 | Sb-02 | 65999614 | 4.7-7.3 | 5.4- 8.2 | 5.9-7.4 | 2, 3, & 5 |
|  | S6_48187126 | Sb-06 | 48187126 | 3.4-5.1 | 4.1- 5.9 | 3.3-8.3 | 1, 4, 5, & 6 |
| **HM** | S1_1535802 | Sb-01 | 1535802 | 3.7-5.3 | 4.5- 6.2 | 2.8-6.3 | 1, 2, 3, & 6 |
|  | S6_48187126 | Sb-06 | 48187126 | 4.3-8.5 | 5.1- 9.4 | 4.8-16.1 | 1, 4, 5, & 6 |
| **YSI** | S1_19966820 | Sb-01 | 19966820 | 3.0-4.3 | 4.0- 5.1 | 3.3-9.5 | 2, 4, & 5 |
| **YL** | S1_17106689 | Sb-01 | 17106689 | 3.1-5.1 | 4.0- 5.9 | 0.5-3.7 | 3, 4, & 5 |
|  | S4_51753172 | Sb-04 | 51753172 | 3.1-5.9 | 4.0- 6.8 | 1.1-8.2 | 2, 4, 5, & 6 |
|  | S7_58489245 | Sb-07 | 58489245 | 3.2-4.5 | 4.0- 5.2 | 1.0-6.7 | 2, 4, & 6 |

Methods 1–6 include 1=mrMLM, 2=FASTmrMLM, 3=FASTmrEMMA, 4=pLARmEB, 5=pKWmEB, and 6=ISIS EM-BLASSO. r ^2^ (%) the proportion of total phenotypic variance explained by each QTN.
