# Supplementary material for: Multi‐locus genome‐wide association study for grain yield and drought tolerance indices in sorghum accessions
Source: Plant Genome. 2024 Sep 10;17(4):e20505. doi: 10.1002/tpg2.20505 (PMC11628898; doi:10.1002/tpg2.20505)
Supplement: Supplementary file 10 — Supplementary Table S7: Candidate genes with significant QTNs identified for drought adaptation in this study. [file TPG2-17-e20505-s003.docx]

**Supplementary Table S7:** candidate genes with significant QTNs identified for drought adaptation in this study

| **QTNs** | **No.** | **Gene Name** | **Description** | **Chr** | **Gene Start (bp)** | **Gene End (bp)** |
| --- | --- | --- | --- | --- | --- | --- |
| S1_1535802 | 1 | Sobic.001G017700 | Similar to Diphthamide biosynthesis protein 2 | Chr01 | 1526937 | 1535046 |
|  | 2 | Sobic.001G017000 | Similar to Putative plastid ribosomal protein S6 | Chr01 | 1496731 | 1498270 |
|  | 3 | Sobic.001G018350 | Predicted protein | Chr01 | 1565061 | 1567644 |
|  | 4 | Sobic.001G018900 | Similar to 40S ribosomal protein S4 | Chr01 | 1596224 | 1598865 |
|  | 5 | Sobic.001G017100 | Similar to Salt tolerance protein | Chr01 | 1500569 | 1503758 |
|  | 6 | Sobic.001G016800 | (1 of 1) K11271 - sister chromatid cohesion protein DCC1 (DSCC1) | Chr01 | 1473756 | 1476285 |
|  | 7 | Sobic.001G018700 | Predicted protein | Chr01 | 1586065 | 1587543 |
|  | 8 | Sobic.001G017400 | Similar to Ras-related protein Rab11C | Chr01 | 1516895 | 1518171 |
|  | 9 | Sobic.001G018300 | Similar to Cytochrome P450 71E1 | Chr01 | 1561988 | 1563879 |
|  | 10 | Sobic.001G018800 | Similar to 60S ribosomal protein L144 | Chr01 | 1590582 | 1592523 |
|  | 11 | Sobic.001G018400 | Weakly similar to Putative uncharacterized protein | Chr01 | 1569993 | 1571101 |
|  | 12 | Sobic.001G017500 | Similar to Putative uncharacterized protein | Chr01 | 1518760 | 1520740 |
|  | 13 | Sobic.001G018500 | Similar to Putative uncharacterized protein OSJNBa0075M12.11 | Chr01 | 1576588 | 1580189 |
|  | 14 | Sobic.001G018600 | Similar to Cytochrome P450 71E1 | Chr01 | 1582349 | 1584298 |
|  | 15 | Sobic.001G017900 | Similar to Os10g0360900 protein | Chr01 | 1538752 | 1542523 |
|  | 16 | Sobic.001G018100 | similar to Putative salt-inducible protein kinase | Chr01 | 1549226 | 1555526 |
|  | 17 | Sobic.001G018000 | similar to FAD dependent oxidoreductase family protein, expressed | Chr01 | 1542704 | 1548779 |
|  | 18 | Sobic.001G018200 | similar to Prohibitin | Chr01 | 1555878 | 1558441 |
|  | 19 | Sobic.001G017600 | similar to ProFAR isomerase associated, putative | Chr01 | 1521168 | 1526686 |
|  | 20 | Sobic.001G017300 | similar to Expressed protein | Chr01 | 1510060 | 1514300 |
|  | 21 | Sobic.001G017800 | (1 of 1) PTHR23101//PTHR23101:SF63 - RAB GDP/GTP EXCHANGE FACTOR // SUBFAMILY NOT NAMED | Chr01 | 1535394 | 1538843 |
|  | 22 | Sobic.001G017200 | similar to Translocation protein-related-like | Chr01 | 1504094 | 1509549 |
|  | 23 | Sobic.001G016900 | similar to RNA-binding protein Luc7-like 2, putative%252C expressed | Chr01 | 1490983 | 1496615 |
| S1_67598132 | 1 | Sobic.001G345333 |  | Chr01 | 67540157 | 67540834 |
|  | 2 | Sobic.001G345366 |  | Chr01 | 67541520 | 67544291 |
|  | 3 | Sobic.001G345400 | similar to Putative ring-H2 zinc finger protein | Chr01 | 67541758 | 67543204 |
|  | 4 | Sobic.001G345600 | (1 of 17) PF04640 - PLATZ transcription factor (PLATZ) | Chr01 | 67578654 | 67582462 |
|  | 5 | Sobic.001G345700 | (1 of 17) PF04640 - PLATZ transcription factor (PLATZ) | Chr01 | 67634187 | 67638202 |
|  | 6 | Sobic.001G345800 | weakly similar to Os09g0116100 protein | Chr01 | 67643603 | 67646810 |
| S10_11382487 | 1 | Sobic.010G111400 | Predicted protein (homologous with MYB-related-transcription factor 81 *Zea maize)* | Chr10 | 11330855 | 11332277 |
|  | 2 | Sobic.010G111450 | (1 of 65) IPR013763 - Cyclin-like | Chr10 | 11343525 | 11344294 |
|  | 3 | Sobic.010G111500 | Predicted protein | Chr10 | 11402946 | 11404307 |
| S5_54698125 | 1 | Sobic.005G110530 | (1 of 3) PTHR14593:SF5 - WD REPEAT-CONTAINING PROTEIN 11 | Chr05 | 54640120 | 54640820 |
|  | 2 | Sobic.005G110514 | (1 of 3) PTHR23155//PTHR23155:SF618 - LEUCINE-RICH REPEAT-CONTAINING PROTEIN // SUBFAMILY NOT NAMED | Chr05 | 54741964 | 54747441 |
|  | 3 | Sobic.005G110511 | (1 of 6) PTHR23155//PTHR23155:SF641 - LEUCINE-RICH REPEAT-CONTAINING PROTEIN // SUBFAMILY NOT NAMED | Chr05 | 54747717 | 54761542 |
| S6_48187126 | 1 | Sobic.006G098200 | Similar to H0818E04.12 protein | Chr06 | 48138075 | 48143144 |
|  | 2 | Sobic.006G098300 | (1 of 2) PTHR23024//PTHR23024:SF220 - MEMBER OF 'GDXG' FAMILY OF LIPOLYTIC ENZYMES // SUBFAMILY NOT NAMED | Chr06 | 48159417 | 48160415 |
|  | 3 | Sobic.006G098400 | (1 of 2) PTHR23024//PTHR23024:SF220 - MEMBER OF 'GDXG' FAMILY OF LIPOLYTIC ENZYMES // SUBFAMILY NOT NAMED | Chr06 | 48161810 | 48163454 |
|  | 4 | Sobic.006G098500 | Similar to H0818E04.14 protein | Chr06 | 48169525 | 48175441 |
|  | 5 | Sobic.006G098600 | weakly similar to H0818E04.15 protein | Chr06 | 48178519 | 48179653 |
|  | 6 | Sobic.006G098800 |  | Chr06 | 48183477 | 48186588 |
